# Engineering transferrable microvascular meshes for subcutaneous islet transplantation

**DOI:** 10.1038/s41467-019-12373-5

**Published:** 2019-10-10

**Authors:** Wei Song, Alan Chiu, Long-Hai Wang, Robert E. Schwartz, Bin Li, Nikolaos Bouklas, Daniel T. Bowers, Duo An, Soon Hon Cheong, James A. Flanders, Yehudah Pardo, Qingsheng Liu, Xi Wang, Vivian K. Lee, Guohao Dai, Minglin Ma

**Affiliations:** 1 Department of Biological and Environmental Engineering, Cornell University, Ithaca, NY 14853 USA; 2 Division of Gastroenterology & Hepatology, Department of Medicine, Weill Cornell Medical College, New York, NY 10021 USA; 3 Department of Mechanical and Aerospace Engineering, Cornell University, Ithaca, NY 14853 USA; 4 Department of Clinical Sciences, Cornell University, Ithaca, NY 14853 USA; 5 Nancy E. and Peter C. Meinig School of Biomedical Engineering, Cornell University, Ithaca, NY 14853 USA; 6 Department of Bioengineering, Northeastern University, Boston, MA 02120 USA

**Keywords:** Tissue engineering, Type 1 diabetes

## Abstract

The success of engineered cell or tissue implants is dependent on vascular regeneration to meet adequate metabolic requirements. However, development of a broadly applicable strategy for stable and functional vascularization has remained challenging. We report here highly organized and resilient microvascular meshes fabricated through a controllable anchored self-assembly method. The microvascular meshes are scalable to centimeters, almost free of defects and transferrable to diverse substrates, ready for transplantation. They promote formation of functional blood vessels, with a density as high as ~220 vessels mm^-2^, in the poorly vascularized subcutaneous space of SCID-Beige mice. We further demonstrate the feasibility of fabricating microvascular meshes from human induced pluripotent stem cell-derived endothelial cells, opening a way to engineer patient-specific microvasculature. As a proof-of-concept for type 1 diabetes treatment, we combine microvascular meshes and subcutaneously transplanted rat islets and achieve correction of chemically induced diabetes in SCID-Beige mice for 3 months.

## Abstract (web summary)

The success of engineered tissue depends on the integration of a dense vascular network to supply nutrients and remove waste products. Here the authors design high density microvascular meshes made through an anchored self-assembly mechanism, and use these meshes to support subcutaneous pancreatic islet survival in a mouse diabetes model.

## Introduction

Vasculature is an essential component of almost any tissue or organ^1^ and therefore vascular regeneration is critical to the success of bioengineered implants^2–7^. For example, in cell replacement therapies for type 1 diabetes (T1D), transplanted insulin producing cells rely on nearby vasculature to survive and function^8–14^. Vascular endothelial cells such as human umbilical vein endothelial cells (HUVECs) can spontaneously assemble into tubular structures in an extracellular matrix (ECM) such as fibrin; however, the structures are usually random, uncontrollable, and less efficient for promoting microvascular regeneration^15^. While conventional molding methods such as seeding cell/matrix mixtures into grooved templates may guide self-assembly into specific patterns^16,17^, such structures tend to shrink and clump during maturation due to intrinsic contractile forces^18^, making fabrication and scale-up of stable and transferrable vasculature a challenge. Interconnected endothelial lumen structures can also be formed by perfusing endothelial cells in microfluidic channels and lining the cells along channel walls^19^. However, confinement within channels inevitably precludes the transfer of endothelial structure to other substrates or devices, limiting the scope of its applications in transplantation. Recent developments in three-dimensional (3D) printing techniques have provided opportunities to fabricate cellular constructs with embedded vascular structures in a controlled manner^20,21^. However, it remains challenging to print high-resolution, microscale vascular structure that is resilient and transferrable to different substrates for transplantation applications.

In this study, we develop a micropillar-based, anchored self-assembly (ASA) strategy to fabricate controllable, transferrable, and scalable microvascular meshes for potential applications in T1D cell replacement therapies. In the ASA, micropillars guide self-assembly of endothelial cells within a fibrin matrix while serving as anchoring points to prevent cellular structure from shrinkage during vessel maturation, leading to controllable, interconnected, and resilient fibrin-filled tubular structures (i.e. microvascular mesh). By tuning the dimension and arrangement of micropillars, we are able to control the geometry (square, pentagon, hexagon, octagon, spider web-like, microcapillary-like), diameter of the fibrin-filled tubes, and mesh opening of the microvascular mesh. These meshes are scalable to centimeter size (5 × 5 cm) and transferrable to diverse substrates. Upon transplantation, microvascular meshes promote both neovascularization and vascular anastomoses. When attached to a diffusion chamber, the meshes lead to the formation of a functional microvascular network with a density as high as ~220 vessels mm^−2^ around the chamber within the poorly vascularized subcutaneous space of severe combined immune deficiency (SCID)-Beige mice. Furthermore, we demonstrate the feasibility of engineering patient-specific microvasculature using human induced pluripotent stem cell-derived endothelial cells (iPSC-ECs). The microvascular meshes (both HUVECs and iPSC-ECs) significantly improve vascularization of subcutaneously transplanted rat islets and enable a 3-month correction of streptozotocin (STZ)-induced diabetes in SCID-Beige mice, providing a proof of concept for the use of microvascular meshes in cell replacement therapies for T1D and potentially other diseases.

## Results

### Fabrication and function of the microvascular meshes

**Fig. 1:**
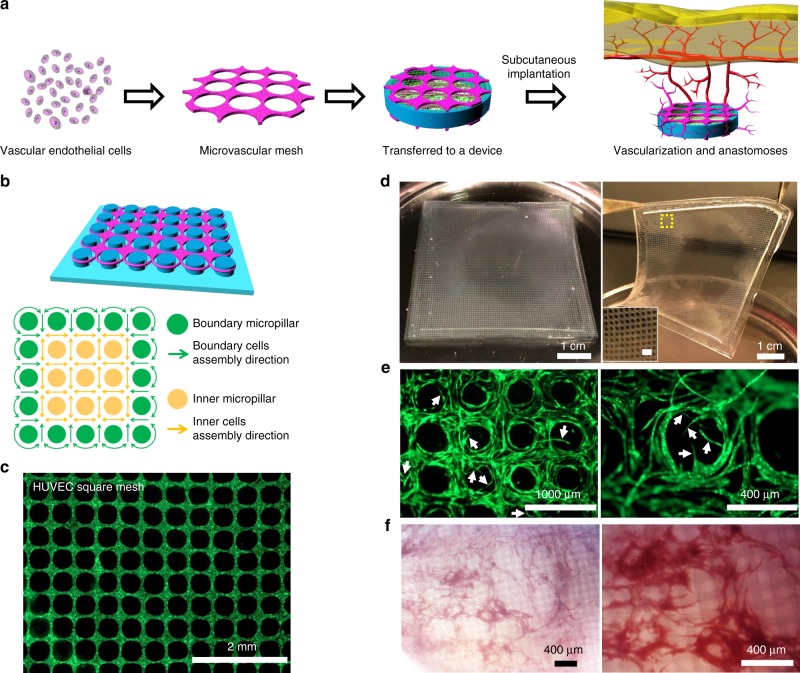
Fabrication and function of transferrable microvascular meshes via anchored self-assembly. **a** Schematic illustration of organization of vascular endothelial cells into a microvascular mesh, which is transferred and attached to a cellular device. In a poorly vascularized subcutaneous space, microvascular mesh can enhance vascularization and anastomoses with host vasculature to provide oxygen/nutrients to donor cells. **b** Design of micropillar arrangement (blue) and ASA-enabled cell organization (purple): the key to the ASA is that inner micropillars (yellow) provide a geometric template for cell self-assembly to form square mesh and boundary micropillars (green) serve as anchoring points for cell attachment to prevent assembled mesh structure from shrinking. **c** A fluorescent image of HUVECs expressing GFP in fibrin matrix organized into a square mesh (1 × 1 cm) on a micropillar substrate via ASA after 2 days of culture. **d** After 2 days of culture, a HUVEC square mesh (5 × 5 cm) is lifted from the micropillar substrate for transfer. The insert is a magnified image of HUVEC mesh and scale bar is 1 mm. **e** HUVEC microvascular mesh generates angiogenic sprouts (white arrows) when embedded in fibrin matrix during 2 weeks of culture in vitro. **f** Microscopic images of retrieved HUVEC microvascular mesh device show a high degree of vascularization after 2 weeks of subcutaneous implantation in SCID-Beige mice (*n* = 4)

To achieve rapid and functional vascularization around a cellular device, one straightforward approach is to attach a pre-formed vascular structure with sufficient density and resolution to the device; upon implantation (e.g. in subcutaneous space) the vascular structure induces angiogenesis and promotes anastomoses with host vasculature (Fig. 1a). To fabricate such a transferrable vascular structure in a controlled manner, we developed an ASA strategy. The key to the ASA is a cell organization process on the micropillar substrate where the inner micropillars serve as a geometric template to guide the cell organization into long-range ordering and the boundary micropillars play an anti-contraction effect to prevent the shrinkage of the mesh structure, leading to a stable and ordered microvascular mesh (Fig. 1b). We found that HUVECs, together with a fibrin matrix, self-assembled into an almost defect-free square mesh after 2 days of culture on the micropillar substrate (Fig. 1c). In contrast, HUVEC structures remained random when cultured on a smooth substrate without micropillars (Supplementary Fig. 1). By adjusting the size and arrangement of micropillars, we could precisely control the mesh geometry and dimension. We illustrated simple geometries such as square, pentagon, hexagon, and octagon (Supplementary Fig. 2a) as well as complex structures that resembled spoke, spider web, and natural capillary bed (Supplementary Fig. 2b). Using square network as an example, we also showed that the diameter of fibrin-filled tubular structure could be controlled from approximately 15 to 133 μm, the size of mesh opening from 135 to 467 μm, and density from 2 to 44 openings mm^−2^ (Supplementary Fig. 2c and Supplementary Table 1).

The ASA approach is scalable and the microvascular meshes can be lifted from the micropillar substrate for transferring. We were able to fabricate a 5 × 5 cm, free-standing, square mesh from HUVECs (Fig. 1d and Supplemental Movie 1). The meshes were stable and cells were viable on micropillar substrate for at least 4 weeks (Supplementary Fig. 3a, b) but rapidly generated sprouts after being transferred and embedded in a fibrin matrix (Fig. 1e and Supplementary Fig. 3c). The meshes promoted dense, functional vascularization on a diffusion chamber after 2 weeks of subcutaneous implantation in SCID-Beige mice (Fig. 1f). Blood-perfused vessels maintained square-like network with numerous newly formed sprouts, similar to angiogenic sprouting in vitro. It should be noted that microvascular meshes were constantly re-molded during the development of vascularization and anastomoses, and therefore the original shapes of mesh network were not always preserved.

### Simulation and characterization of the microvascular meshes

**Fig. 2:**
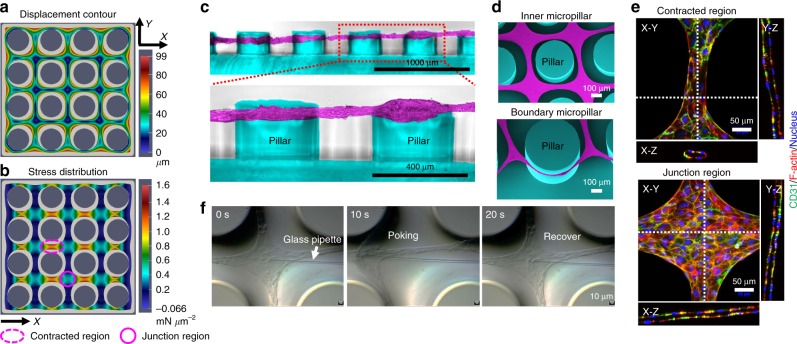
Simulation and characterization of the ASA-enabled microvascular meshes. **a**, **b** The contraction simulation shows an in-plane displacement contour plot of organized cellular mesh structure (**a**) and the normal stress distribution in the *X* (Cauchy stress component 11) direction (**b**) on a 4 × 4 micropillar substrate. The initial shape of cells and fibrin matrix is displayed in light gray. The micropillar diameter is 400 μm and micropillar-to-micropillar interval is 200 μm. The contracted region is marked as dotted purple ellipse and the junction region is purple circle. The displacement unit is μm and the unit of stress is mN μm^−2^. **c** Cross-sectional images showing a HUVEC mesh suspended between micropillars. The micropillars are pseudo-colored as blue and HUVECs are pseudo-colored as purple. **d** SEM images of a HUVEC mesh (purple) at the inner and boundary regions on the micropillar substrate (blue). **e** Confocal images of a HUVEC mesh at the contracted and junction regions on the micropillar substrate showing the tubular structures. Human CD31 antibody is green, F-actin is red, and nucleus is blue. **f** Screenshots of a glass pipette poking a HUVEC mesh showing high resilience of the mesh

To better understand the ASA process, we performed a finite element simulation of the cellular assembling process and further characterization of the microvascular mesh. The simulation, which considers the contractile action of the cells on the fibrin matrix, generated an in-plane displacement contour plot of organized mesh structure (Fig. 2a) and stress and strain distribution (Fig. 2b and Supplementary Fig. 4) on the micropillar substrate. The assembling cells and matrix gradually stopped being in contact with the inner micropillars, and finally only the boundary micropillars were in contact with cells and supported the entire mesh structure (Fig. 2a). The cells and matrix close to boundary micropillars sustained contraction from one direction, while those away from the boundary experienced contraction from all directions, and exhibited higher stresses in the contracted region than in the junction region along both the *X* (Cauchy stress component 11 in Fig. 2b) and *Y* (Cauchy stress component 22 in Supplementary Fig. 4a) directions.

Interestingly, cross-sectional images showed that indeed the microvascular meshes were tightened and suspended between micropillars rather than settling at the bottom (Fig. 2c), consistent with the simulation results. Scanning electron microscopic (SEM) images (Fig. 2d and Supplementary Fig. 5) also confirmed that the HUVEC mesh hung among inner micropillars with more contracted regions between junctions and the whole mesh was prevented from shrinking by boundary micropillars. Control experiments further supported that the formation of a stable cell construct is not through a simple space-filling mechanism alone but highly relying on the micropillars. For example, when HUVEC/fibrin mixture was introduced into grooves with different shapes (e.g., linear, triangle, cross, and windmill) without micropillars inside, cell/fibrin mixture formed structures that were only temporarily stable and all shrank into clumps within 48 h (Supplementary Figs. 6a and 7a) due to intrinsic cellular contraction. In contrast, when micropillars were present inside, cells self-organized into different structures that corresponded to the shapes of the grooves (Supplementary Figs. 6b and 7b).

Confocal images showed that the HUVEC mesh (approximately 25 μm thick after 2 days of culture) had continuous and interconnected tubular structures (Fig. 2e and Supplementary Fig. 8a) in both contracted and junction regions. Further staining showed that the interior of the tubular structure was filled with fibrin on which HUVECs coalesced and adhered (Supplementary Fig. 8b). This self-assembled, cell/fibrin composite structure was consistent with earlier reports^6,22^ and resembled the de novo formation of primitive vasculatures that also involves coalescence of endothelial progenitor cells and subsequent lumen formation^23,24^. Another important characteristic of the ASA-enabled microvascular meshes is their mechanical robustness. The meshes were elastic and resilient; they even withstood poking with a 6-μm glass pipette. As shown in Fig. 2f and Supplemental Movie 2, the mesh was displaced approximately 150 μm without any visible damage and then recovered to its original position when the pipette was withdrawn. This remarkable mechanical property allowed us to manipulate and transfer the mesh to different substrates (Supplementary Fig. 9) without affecting the integrity and fibrin-filled tubular structures of the mesh (Supplementary Fig. 10).

### Enhanced vascularization of subcutaneous devices in SCID-Beige mice

**Fig. 3:**
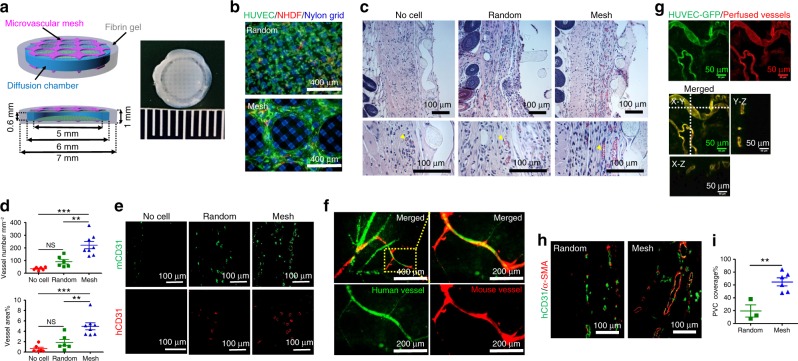
Enhancement of vascularization and anastomoses in subcutaneous space of SCID-Beige mice. **a** Schematics and a digital photo of a Mesh device, which is a diffusion chamber with HUVEC meshes (~25 μm thick, purple) in the fibrin gel (gray) on the top and bottom. The diffusion chamber is a cylindrical cell container with a PDMS ring (blue) as the wall and two nylon grids (green) as the top and bottom. The dimensions of each component are labeled in the schematic. **b** Fluorescent images of randomly mixed cells (top) and microvascular mesh (bottom) placed on diffusion chambers after 2 days of culture in EGM-2 medium. HUVEC:NHDF = 9:1, Human CD31 antibody is green to show HUVEC, α-smooth muscle actin (α-SMA) antibody is red to show NHDF, and nylon grid is blue. **c** Cross-sectional hematoxylin/eosin staining images of retrieved devices after 14 days of implantation. Yellow arrowheads point to blood vessels with erythrocytes inside. **d** Density and area percentage of blood vessels at the interface between the device and panniculus carnosus muscle. *n* = 6 in the No cell and Random, and *n* = 8 in Mesh groups. Data are mean ± SEM; **P < 0.01, ***P < 0.001, NS (*P* > 0.05) no significant difference. One-way analysis of variance. **e** Cross-sectional immunostaining images of human (red) and mouse (green) CD31 antibodies showing the human and mouse blood vessels at the interface between the device and panniculus carnosus muscle. **f** Confocal images of perfused lectins bound to human (UEA-I, green) and mouse (GSL-I, red) endothelial cells, confirming the anastomoses between human and mouse vessels. **g** Blood-perfused human vasculatures anastomosed with mouse vascular system in Mesh device after 10 days of subcutaneous implantation. HUVEC-GFP is green and perfused dye DiI in vessels is red. **h** Representative immunostaining images of mature human vasculatures (human CD31 antibody is green) covered with perivascular cells (α-SMA antibody is red) in retrieved Random and Mesh device after 10 days of subcutaneous implantation. **i** The percentage of perivascular cell (PVC) coverage is 19 ± 9% (*n* = 3; mean ± SEM) and 65 ± 6% (*n* = 6) for the Random and Mesh devices, respectively. **P < 0.01. Unpaired two-tailed *t* test

To quantitatively investigate how microvascular meshes enhanced vascularization, we compared HUVEC meshes with random HUVEC/fibrin mixture. In both cases, normal human dermal fibroblasts (NHDFs) were added (HUVECs:NHDFs = 9:1) to support and enhance vessel formation^25^. Microvascular meshes or random cell mixture were attached to diffusion chambers using a fibrin gel (Mesh device (*n* = 8) and Random device (*n* = 6); Fig. 3a, b; details in Methods and Supplementary Fig. 11). Devices without any cells (No cell device (*n* = 6)) were used as an additional control. All devices were then implanted into subcutaneous space of SCID-Beige mice. The subcutaneous space is a poorly vascularized site but has many advantages for cell replacement therapies including relatively easy accessibility, minimal invasiveness, and potentially high transplant capacity^26^.

The devices were retrieved, and vascularization was compared after 2 weeks of implantation. Histological hematoxylin/eosin (H&E) staining (Fig. 3c) and quantification of blood vessels (Fig. 3d) surrounding the chambers revealed a significantly higher vascularization in the Mesh device, compared to the No cell and Random devices. These vasculatures were covered by perivascular cells (PVCs) as indicated by α-smooth muscle actin (α-SMA) staining (Supplementary Fig. 12). Interestingly, positive immunostainings for both human CD31 (Fig. 3e; red) and mouse CD31 (Fig. 3e; green) seemed to suggest that newly formed vessels (in both Random and Mesh devices) were chimeric in nature, indicating the occurrence of anastomoses and vascular re-modeling during vascularization. To further demonstrate that generated vessels were functionally connected with mouse vasculature and to confirm the presence of anastomoses, we perfused mice with the Mesh devices through tail vein injections using two lectins. The first lectin was a green fluorescent *Ulex europaeus* Agglutinin I (UEA-I) lectin that specifically bound to human ECs^6^ and the second one was a red *Griffonia simplicifolia* lectin I (GSL-I) isolectin B_4_ that was specific for mouse ECs^27^. The overlap of human- and mouse-specific lectin binding (Fig. 3f) supported that HUVEC mesh promoted not only neovascularization but also anastomoses. In addition, to evaluate the earliest time point that blood-perfused human vasculatures were formed and anastomosed with mouse vascular system, we subcutaneously implanted microvascular meshes of HUVEC-GFP/NHDF (9:1, Mesh device) and random mixture of HUVEC-GFP/NHDF (9:1, Random device) and perfused lipophilic carbocyanine dye DiI^28^ into mouse vascular system at different time points (Days 4, 7, and 10, Supplementary Fig. 13 and Fig. 3g). At Day 10, in Mesh device, we found that human vasculatures were functional and connected with mouse vascular system since green color of HUVEC-GFP overlapped with red color of perfused dye DiI. However, in Random device, we did not observe the formation of blood-perfused human vessels under the confocal microscope (Supplementary Fig. 13a). The image analysis showed that the percentage of blood-perfused human vasculatures is 50.4 ± 15.6% (*n* = 5; mean ± SEM) in Mesh devices (Supplementary Fig. 13b). Furthermore, histological slides of retrieved Random and Mesh devices were stained with human CD31 and α-SMA antibodies. The vasculatures covered by PVCs were identified by vessel cross-sections, which had luminal shape (with erythrocytes inside) and were positively stained by both human CD31 and α-SMA antibodies (Fig. 3h). The percentage of PVC coverage is 19 ± 9% (*n* = 3) in Random device and 65 ± 6% (*n* = 6) in Mesh device (Fig. 3i). All the results, taken together, substantiate that ASA-enabled, transferrable microvascular meshes have potential use for vascularization of cell delivery devices.

### Correction of diabetes in SCID-Beige mice using rat islets

**Fig. 4:**
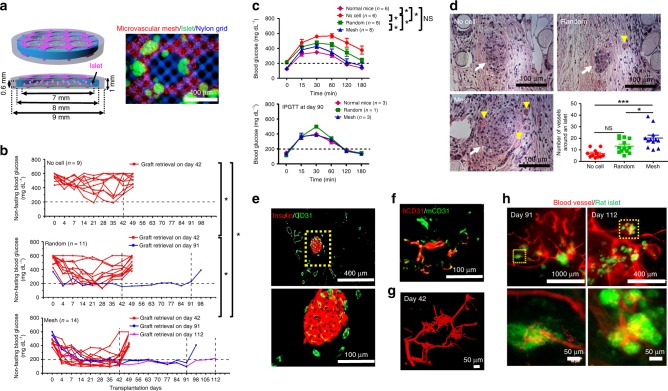
Improvement of re-vascularization of rat islets and diabetes correction in SCID-Beige mice. **a** Schematics and a microscopic image of rat islets (green) in a Mesh device. Microvascular mesh is red and nylon grid is blue. **b** Non-fasting blood glucose (BG) concentration of the mice after transplantation. Grafts were retrieved after different time points. Most of the mice were kept alive for 1 week after retrieval while 3 mice from the Mesh group were used for perfusion studies prior to retrievals. During 42 days of transplantation (*n* = 9 in No cell, *n* = 11 in Random, and *n* = 14 in Mesh): *P < 0.05 and NS (*P* > 0.05) no significant difference. ANCOVA, time was treated as continuous covariate. **c** BG concentrations during intraperitoneal glucose tolerance tests (IPGTT) after 30 and 90 days of transplantation. IPGTT at Day 30 (*n* = 6 in Normal mice and No cell, *n* = 8 in Random and Mesh). Data are mean ± SEM. *P < 0.05 and NS (*P* > 0.05) no significant difference. ANCOVA, time was treated as continuous covariate. **d** Hematoxylin/eosin staining of rat islets and blood vessels in retrieved devices (Day 42) and the number of blood vessels around a rat islet (within a 200 μm distance). Yellow arrowheads point to blood vessels with erythrocytes inside. White arrows point to rat islets. The round material in the cross-sections is nylon grid. No cell group consists of 11 islets pooled from 3 mice; Random group consists of 13 islets pooled from 3 mice; Mesh group consists of 12 islets pooled from 3 mice. *P < 0.05, ***P < 0.001, and NS (*P* > 0.05) no significant difference. One-way analysis of variance. **e** Cross-sectional immunostaining images of rat insulin (red) and mouse blood vessels (CD31, green) in a retrieved Mesh device. **f** Immunostaining images (parallel section) using human (red) and mouse (green) CD31 antibodies indicate the anastomoses between human and mouse blood vessels. **g** Confocal image of perfused blood vessels in re-vascularized rat islets in the Mesh group after 42 days of transplantation. **h** Fluorescent images of re-vascularized rat islets after 91 and 112 days of transplantation from the Mesh group. The rat islets expressing GFP are green and perfused blood vessels are red

We next investigated whether microvascular meshes could be used to improve cell replacement therapy for T1D. We loaded rat islets in diffusion chambers (Fig. 4a; more details in Methods and Supplementary Fig. 14), attached microvascular meshes (HUVECs:NHDFs = 9:1) to the chambers using a fibrin gel (Mesh device), and transplanted the constructs subcutaneously in SCID-Beige mice with STZ-induced diabetes. The chamber had an open structure with pore size ~70 μm, smaller than the average islet size (~150 μm). Each device contained ~500 islet equivalents (IEQ)^29^. To better visualize islets, we loaded green fluorescent protein (GFP) rat islets in two Mesh devices (Fig. 4a). Devices encapsulating similar number of islets but with random HUVECs/NHDFs (Random device) or without any HUVECs/NHDFs (No cell device) were used as controls. After transplantation, non-fasting blood glucose (BG) concentration decreased in some mice in all three groups. It is worth noting that non-fasting BG may be affected by the timing of food intake. While it is possible that a lower BG may be the result of the mouse not eating prior to BG measurement, comparison among different groups still showed significant differences in BG control. Mice in the Mesh group (*n* = 14) had significantly better BG control compared to the No cell (*n* = 9) and Random groups (*n* = 11) during 42 days of transplantation (Fig. 4b and Supplementary Table 2). Although the earliest formation of blood-perfusable, anastomosed vasculature was observed on Day 10 in empty Mesh device (no islet, Fig. 3g and Supplementary Fig. 13), 4 of the 14 mice in the Mesh group had normal BG as early as 4 days posttransplantation (Supplementary Table 2). This may suggest that the viability and function of islets could benefit from the paracrine secretion/signaling of HUVEC microvascular meshes during early days of transplantation. The release of insulin from some dying islets might also assist in the early normalization of BG. The number of normoglycemic mice in the Mesh group increased to 10 at 14 days posttransplantation (Supplementary Table 2) after functional human vasculatures and anastomoses with mouse vascular system had been established. In the Random group, 5 of the 11 mice showed normoglycemia at 21 days; however, the number of normoglycemic mice decreased to 3 at 28 days. Although there was a mouse being able to control normal BG for 90 days, other mice in the Random group were hyperglycemic after 35 days. The randomly dispersed individual HUVECs required a longer time to proliferate, coalesce to form tubular structures, and induce the ingrowth of host vasculatures and therefore might only support the survival and function of a fraction of islets.

Grafts were retrieved for characterization at Day 42 from most mice except 1 normoglycemic mouse from the Random group and 3 normoglycemic mice from the Mesh group, which were kept longer until Day 91 or Day 112. After retrieval, the BG increased in all mice, demonstrating the anti-diabetic effect of the devices. We also performed an intraperitoneal glucose tolerance test (IPGTT) at 30 and 90 days posttransplantation (Fig. 4c). At 30 days, compared to the No cell and Random groups, mice in the Mesh group showed better BG control and tolerance during the stimulation of injected glucose. At 90 days, since the remaining mice had a small number and were all normoglycemic, IPGTT showed no difference between the groups.

The devices retrieved at Day 42 were evaluated histologically for vascularization (Fig. 4d). Although viable islets with normal morphology were found in all the three groups, islets in implants from the Mesh group were surrounded by significantly more blood vessels than the two control groups, consistent with the diabetes correction results (Fig. 4b). Although NHDFs were incorporated with HUVECs to promote the maturation of functional vessels, we still noticed that a few erythrocytes leaked out of immature vessels (hemorrhage) in histological staining. To further enhance the maturation of newly formed vessels, mesenchymal stem cells could be used as supporting cells^30^. Additional immunostaining (Fig. 4e) confirmed that islets in the Mesh group were functional with positive insulin staining and were also highly vascularized (CD31 staining) both externally and internally. Similar to the results with empty devices (no islet, Fig. 3e, f), positive overlapping staining with both human and mouse CD31 antibodies demonstrated anastomoses between human and mouse vessels (Fig. 4f). Moreover, to better visualize the connection between vasculatures inside rat islets and surrounding mouse vessels in mice from the Mesh group, we performed whole-mouse perfusions, prior to retrievals at Day 42, Day 91, and Day 112, with a fluorescent lipophilic carbocyanine dye DiI^28^. Confocal and fluorescent images of the perfused devices clearly showed interconnected, 3D structure of vasculatures in transplanted islets (Fig. 4g, Supplementary Fig. 15, and Supplemental Movie 3 for the device from Day 42 retrieval and Fig. 4h for the device with GFP rat islets from Day 91 and Day 112 retrievals). Together, these results confirmed the effectiveness of transferrable microvascular meshes in promoting re-vascularization of donor islets and maintaining normoglycemia for up to 3 months in diabetic mice.

### Microvascular meshes from human iPSC-ECs

**Fig. 5:**
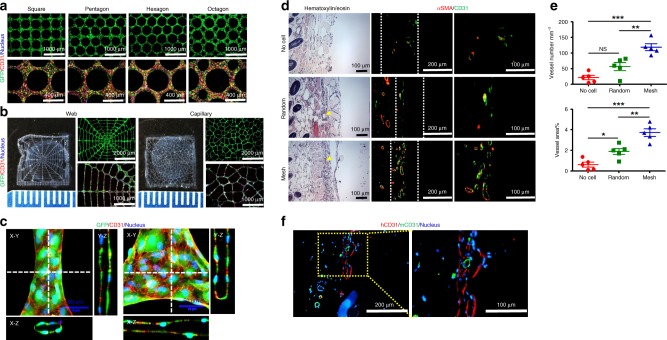
Human iPSC-EC-derived microvascular meshes enhance vascularization. **a** Fluorescent images of iPSC-EC meshes with different geometries (square, pentagon, hexagon, and octagon). **b** iPSC-EC meshes with more complex patterns (spider web and capillary bed). **c** Confocal images of an iPSC-EC mesh at the contracted and junction regions. iPSC-EC expressing GFP is green, human CD31 antibody is red, and nucleus is blue. All samples were imaged after 2 days of culture. **d** Hematoxylin/eosin and immunostaining images of retrieved devices after 2 weeks of subcutaneous implantation in SCID-Beige mice. In all in vivo experiments, NHDFs were mixed with iPSC-ECs (iPSC-EC:NHDF = 9:1). The yellow arrowheads point to blood vessels with erythrocytes inside. The white dash lines mark the interface between the device and panniculus carnosus muscle. Mouse CD31 antibody is green and α-smooth muscle actin (α-SMA) is red. **e** The density and area percentage of blood vessels at the interface. *n* = 5 in all groups. Data are mean ± SEM. *P < 0.05, **P < 0.01, ***P < 0.001, and NS (*P* > 0.05) no significant difference. One-way analysis of variance. **f** Immunostaining images of human (red) and mouse CD31 (green) antibodies showing the anastomoses between iPSC-EC derived vessels and mouse vessels

To explore whether the ASA strategy was applicable to other types of ECs and would be potentially used in a clinical setting, we tested human iPSC-ECs. iPSC-ECs have been considered as an autologous, unlimited cell source for vascularization^31^ and therefore have great potential for clinical applications. Similar to HUVECs, iPSC-ECs formed various controllable microvascular meshes with fibrin-filled tubular structures on micropillar substrates (Fig. 5a–c). The different geometrical meshes can be transferred to diffusion chambers (Supplementary Fig. 16). To evaluate the ability to enhance vascularization, microvascular meshes of square shape were implanted in subcutaneous space of SCID-Beige mice for 2 weeks. In all in vivo experiments, a small amount of NHDFs were mixed with iPSC-ECs (iPSC-ECs:NHDFs = 9:1). As shown in Fig. 5d, H&E staining and immunostaining (CD31 and α-SMA) revealed vasculatures covered by PVCs at the interface between the device and panniculus carnosus muscle for all the three groups. However, the Mesh devices (*n* = 5) resulted in significantly more blood vessels in terms of both density and area percentage than the No cell (*n* = 5) and Random (*n* = 5) devices (Fig. 5e). Moreover, the formation of blood-perfused iPSC-EC vessels was observed on Day 10 posttransplantation, and the percentage of blood-perfused iPSC-EC-derived human vasculatures was 47.0 ± 20.3% (*n* = 3). (Supplementary Fig. 17). Positive staining of human and mouse CD31 antibodies confirmed the anastomoses (Fig. 5f).

**Fig. 6:**
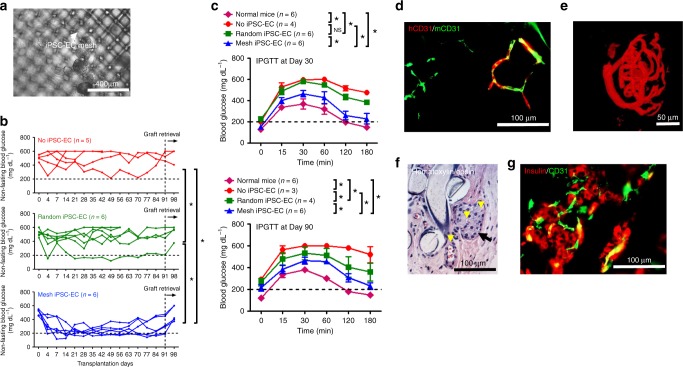
Improved diabetes correction by the iPSC-EC microvascular meshes in SCID-Beige mice. **a** A microscopic image of rat islets in an iPSC-EC mesh device. The gray color aggregates are rat islets. The white arrow points to iPSC-EC mesh. In all in vivo experiments, NHDFs were mixed with iPSC-ECs (iPSC-EC:NHDF = 9:1). **b** Non-fasting BG concentration of the mice during 91 days of subcutaneous transplantation (*n* = 5 in No iPSC-EC, *n* = 6 in Random iPSC-EC and Mesh iPSC-EC). *P < 0.05. ANCOVA, time was treated as continuous covariate. **c** BG concentration during intraperitoneal glucose tolerance test (IPGTT) after 30 and 90 days of transplantation. IPGTT at Day 30 (*n* = 6 in Normal mice, Random iPSC-EC, and Mesh iPSC-EC, and *n* = 4 in No iPSC-EC). Data are mean ± SEM. *P < 0.05. NS (*P* > 0.05) no significant difference. IPGTT at Day 90 (*n* = 6 in Normal mice, *n* = 3 in No iPSC-EC, *n* = 4 in Random iPSC-EC, and *n* = 6 in Mesh iPSC-EC): *P < 0.05. ANCOVA, time was treated as continuous covariate. **d** Immunostaining images (parallel section) of human (red) and mouse CD31 (green) antibodies indicating the anastomoses between human and mouse blood vessels. **e** Confocal image of perfused blood vessels in a re-vascularized rat islet retrieved from the Mesh group at Day 91. **f** Hematoxylin/eosin staining image of a retrieved iPSC-EC mesh device at Day 91. Black arrow points to a rat islet and yellow arrowheads point to blood vessels containing erythrocytes. **g** Immunostaining image of insulin and blood vessels surrounding rat islets in a retrieved iPSC-EC mesh device at Day 91. Rat insulin antibody is red and mouse CD31 antibody is green

The iPSC-EC mesh was attached to a diffusion chamber containing rat islets using a fibrin gel (Mesh iPSC-EC (*n* = 6); Fig. 6a) similar to HUVEC mesh. The Mesh group led to significantly better diabetes correction than the control groups (No iPSC-EC (*n* = 5) and Random iPSC-EC (*n* = 6)) in the rat-to-mouse transplantation model (Fig. 6b and Supplementary Table 3). Mice from the Mesh group also responded to IPGTTs, performed on Day 30 and Day 90, significantly better than those from control groups (Fig. 6c). Compared to normal mice, the glucose response of mice in Mesh group was delayed. In the future, enhancing the function and vascularization of iPSC-EC mesh might help transplanted islets realize improved BG control and glucose responsiveness in IPGTT. Immunostaining of the retrieved Mesh devices confirmed that iPSC-EC meshes promoted anastomoses between human and mouse vessels (Fig. 6d). Whole-mouse perfusion prior to retrieval and confocal imaging showed that the transplanted rat islets were functionally re-vascularized (Fig. 6e and Supplemental Movie 4). Re-vascularization and insulin secretion by the islets were verified by H&E (Fig. 6f) and immunostaining (Fig. 6g). Blood-perfused vessels were present both inside and outside the islets (H&E image), and the islets were positive for both insulin and CD31 staining. These results demonstrate the feasibility of engineering patient-specific microvascular meshes from iPSC-ECs and the potential use in cell replacement therapies for T1D.

## Discussion

We utilize spatially arranged micropillars to fabricate high-resolution, resilient, and transferrable microvascular meshes. Micropillars play two roles in the ASA: guiding the ECs to form patterns with controllable geometry and preventing the cells and matrix from shrinking. Compared to random EC tubes formed on a smooth substrate, the ASA-enabled microvascular meshes are continuous, interconnected, and precisely controlled. In a poorly vascularized subcutaneous space, microvascular meshes promote more efficient vascularization and anastomoses with host vasculature than randomly mixed cells. Subcutaneous space is an attractive transplant site due to its easy accessibility and relatively large capacity for transplantation^26^; however, subcutaneous space has much less vasculature^32,33^ compared to other vascularized sites, such as small bowel mesentery^13^, omentum^34^, and epididymal fat pad^35^. In Random device, cells were homogeneously dispersed inside the fibrin gel on top and bottom of the diffusion chamber and required time to form interconnected network. In contrast, microvascular meshes provide a pre-formed, highly interconnected network for secretion of angiogenic factors and generation of vascular sprouts. The hierarchical structures of pre-formed mesh network and newly branched sprouts efficiently induce ingrowth and anastomoses of host vasculature. Likely due to such multilevel configuration, microvascular meshes resulted in a high vasculature density. Our results are consistent with earlier work showing that HUVEC organization in vitro can impact anastomoses in vivo. For example, Levenberg et al. showed that single HUVECs cultured only 1 day in vitro formed blood-perfused vessels after 14 days of transplantation in subcutaneous space, whereas anastomoses happened after 10 days of transplantation of HUVECs that were cultured for 14 days and organized into vascular-like networks^36^. In this study, we focused on the square microvascular mesh and compared its vascularization with random cells on the smooth substrate without any micropillars. Given that square shape might not be the optimal one, future research will be required to compare the effects of different microvascular mesh opening (controlled by micropillar size/interval) and geometry (controlled by micropillar arrangement) on the vascularization. In addition, we chose the SCID-Beige mice as recipients to avoid the compounding effect of immune rejections, similar to most previous studies^8,9,12^. However, this strain is also known to promote angiogenesis. Immunocompetent animals and autologous endothelial and supporting cells should be tested in future to show the translational potential of our approach.

The microvascular mesh although thin is within the same size range of capillary in vivo (~5–40 μm) and the network structure mimics the capillary bed in vivo and is able to enhance vascularization. The work by Chen’s group^6,24^ also showed that thin vascular structures (i.e., ~50 μm endothelial cell cords) could enhance vascularization. Furthermore, even thicker vascular-like structures fabricated in vitro would significantly shrink and generate thin vasculatures in vivo. For example, it was found that endothelial cord of 500 μm diameter resulted in vessel size of ~15 μm in vivo^22^. We speculate that these thick cellular structures were unstable and not completely contracted in vitro, which caused dramatical size shrinkage in vivo. Interestingly, although the thickness of our microvascular mesh was thin in vitro, the size remained similar upon transplantation based on the observation of the blood-perfused human vasculatures in mice. This might be the result of more complete cellular contraction from our unique fabrication method.

The resilient and transferrable microvascular meshes that we described in this work made it possible to vascularize islets subcutaneously transplanted in retrievable delivery devices. Cell replacement therapy such as intrahepatic transplantation of donor islets is relatively successful in some patients. However, there are a number of limitations, including immediate blood-mediated inflammatory response^37^, potential risks of thrombosis^38^ and localized steatosis^39^, and inability to retrieve or replace failed cells^40^. Therefore, great efforts have been made to find an alternative islet transplantation strategy that is less invasive, supports long-term cell function, and allows cell retrieval or replacement^8,12,40–43^. Previous studies have shown that rat islets subcutaneously transplanted in immunodeficient mice maintained short-term normoglycemia (~20–28 days) at the low dose (~375–750 islets or IEQ), but the long-term islet function was not presented^9,44^. We demonstrated that the microvascular meshes significantly improved the function of rat islets (approximate 500 IEQ) in the poorly vascularized but convenient subcutaneous space and enabled diabetes correction in SCID-Beige mice for up to 3 months. Importantly, given that microvascular meshes may be made from autologous cells such as iPSC-ECs and can be transferred to different delivery or immuno-protective devices, our approach may contribute to an immunosuppression-free cell replacement therapy for T1D.

The scalability is an important requirement for cell replacement therapies and has been challenging for subcutaneous devices^40,45–47^. ASA-enabled microvascular meshes can be fabricated in larger sizes (i.e. ~25 cm^2^) and stacked with alternating devices in the *Z* direction for scale up (Supplementary Fig. 18a, b). High level of vascularization was observed in the stacked construct (Supplementary Fig. 18c), and the vessel density is similar between the top layer and middle layer (Supplementary Fig. 18d). In the middle layer, blood-perfused human vasculatures were formed (Supplementary Fig. 18e), rat islets remained viable (Supplementary Fig. 18f), and the intra-islet vasculatures could be perfused (Supplementary Fig. 18g) after 2 weeks of transplantation. Given that different types of ECs may be isolated from the body^48–50^, patient- and tissue/organ-specific microvascular meshes could be created using the ASA. In this work, we only demonstrated one line of iPSC-EC, but in principle different iPSC-EC lines could be organized into microvascular meshes to explore the broader applicability of ASA-enabled cell self-assembly and vascularization. Moreover, microvascular meshes may be combined with other cell types such as hepatocytes and cardiomyocytes for liver and cardiovascular engineering applications or fibroblasts and smooth muscle cells to assist wound healing. Lastly, the ASA as a general approach may be expanded to other bioengineering fields to construct geometrically defined, high-resolution live materials at micro-scales or to organize therapeutic cells into specific patterns for diverse applications.

## Methods

### Fabrication of micropillar substrate

The micropillar substrate was composed of polydimethylsiloxane (PDMS) and fabricated using standard soft lithography at the Cornell NanoScale Facility. Briefly, a photomask was prepared using a mask writer (DWL2000, Heidelberg Instruments). The silicon wafer was spin-coated with SU-8 100 photoresist (MicroChem) at 500 rpm for 30 s and then 1300 rpm for 30 s. The coated wafer was covered with the photomask and exposed to ultraviolet (UV) light in a UV photolithography machine (ABM Contact Aligner) for 55 s. After being developed and post-baked, the SU-8 master wafer was fabricated. The master wafer contained 200-μm-deep microwells with various geometries. The master wafer was used to create PDMS (Sylgard 184, Dow Corning) micropillars. A mixture (10:1, w–w) of Sylgard 184 silicone elastomer components was casted onto the master wafer, cured at 60 °C overnight, and peeled off from the master to obtain a PDMS micropillar substrate.

### Cell culture

Normal HUVECs (passages 4–6, Lonza) or HUVECs expressing GFP were cultured in Endothelial Cell Growth Medium-2 (EGM-2) (Lonza). NHDFs (passages 4–10, Lonza) were cultured in fibroblast growth factor-2 (FGF-2) medium (Lonza). Cells were plated at a density of ~10,000 cells cm^−2^ and grown at 37 °C and 5% CO_2_ incubator to ~80% confluence over ~6 days prior to experiment. Cells were not tested for mycoplasma contamination in house but Lonza certified them free of contamination.

To differentiate human iPSC into ECs (iPSC-ECs)^51^, embryonic bodies were generated from iPSC (LN4, a subclone of iPS.C2a that was initially reprogrammed from human foreskin fibroblasts^52^) and cultured on non-adherent plates in advanced Dulbecco’s modified Eagle’s medium/F12 medium (Gibco) supplemented with 20% (v/v) knockout serum replacement (Invitrogen), non-essential amino acids (Gibco), L-glutamine (Invitrogen), penicillin/streptomycin (Invitrogen), β-mercaptoethanol (Gibco), and then cytokines were added sequentially: 20 ng mL^−1^ bone morphogenetic protein-4 (R&D Systems) (Days 0–7), 10 ng mL^−1^ Activin A (R&D Systems) (Days 1–4), and 8 ng mL^−1^ FGF-2 (Peprotech) (Days 2–14). On Day 4, embryonic bodies were transferred to adherent conditions on Matrigel (BD Biosciences) coated plates and medium was supplemented with 25 ng mL^−1^ vascular endothelial growth factor-A (Peprotech) (Days 4–14), 10 μM SB431542 (Tocris) (Day 7–remainder of experiment). After 14 days, cell mixtures were dissociated using Accutase (eBioscience) and sorted for CD31^+^ endothelial cells. Purified iPSC-ECs were cultured in EGM-2 medium for further experiment.

### Formation of microvascular meshes by ASA

PDMS micropillar substrates were autoclaved, treated with UV ozone cleaner (Model 18, Jelight) for 10 min, placed in a 24-well plate, and coated with 1% (w/v) Pluronic^®^ F127 (Sigma) solution before cell seeding to prevent cell attachment on PDMS surface and to facilitate cell assembly.

To form microvascular meshes (Supplementary Fig. 11), cells were suspended in fibrin solution (3 mg mL^−1^ fibrinogen (from bovine plasma, Sigma) and 1.0 U mL^−1^ thrombin (from bovine plasma, Sigma)) at a concentration of 8.0 × 10^6^ cells mL^−1^ and poured over a PDMS micropillar substrate. Excess cell suspension was scraped off with a cover glass. The cells in fibrin solution homogeneously filled the interspaces between micropillars. After 15 min of incubation at 37 °C, a fibrin gel was formed and cells embedded inside were cultured in EGM-2 medium. Microvascular meshes formed between micropillars after overnight culture and further stabilized during subsequent 2 days of culture. All the in vitro characterization of microvascular mesh was performed after 2 days of culture. In some experiments, fibrinogen conjugated with Alexa Fluor™ 488 (Molecular Probes, Cat No. F13191) was mixed with normal fibrinogen to form fluorescent fibrin gel (1:200 dilution).

### Simulation

We postulate a free energy that has a passive elastic contribution from the fibrin matrix and an active contractile contribution from the cells, and we consider that they act in parallel. The effects of contractility are assumed to be greater than the poroelastic effects in the contracting microtissue, thus the effect of pore pressure is neglected, essentially considering the fibrin matrix as a Neo-Hookean compressible foam on which the cells are acting. Both of these assumptions are prevalent in the literature for modeling and simulation of contractile microtissues^18,53^, adequate for capturing the key features of the response of microtissues. The assumed free energy has the form

1 $$U\left( {I_{\mathrm{C}},J,\eta _{{\mathrm{iso}}}} \right) = U_p\left( {I_{\mathrm{C}},J} \right) + U_a\left( {J,\eta } \right) = \frac{\mu }{2}\left( {I_{\mathrm{C}} - 3 - 2{\mathrm{ln}}\left( J \right)} \right) + \frac{\lambda }{2}\left( {{\mathrm{ln}}\left( J \right)} \right)^2 \, + \, \eta _{{\mathrm{iso}}}\beta \left( {{\mathrm{ln}}\left( J \right)} \right)^2$$

In the last part of the equation, the first two terms correspond to the compressible Neo-Hookean formulation for the homogenized fibrin network and the last term to the homogenized active contractile action of the cells. Decoupling the energetic contributions is a prevalent assumption in the field of computational models^53^. Considering both the active (cell) and passive (fibrin) parts of the free energy is critical in the prediction of the aggregate response of the composite microtissue. Where $I_C$ is the first principal invariant of the right Cauchy–Green deformation gradient $C_{IJ} = F_{iI}F_{jJ}$, $J$ is the third principal invariant of the deformation gradient $F_{iJ} = \partial x_i/\partial X_J$, where lowercase letters refer to the reference and capital refer to current configuration. The shear modulus of matrix is denoted as *μ* and Lame’s first parameter is *λ*. The cell isotropic contractile activation $\eta _{{\mathrm{iso}}}$ is a dimensionless parameter that ranges from 0 to 1 characterizing the intensity of the cellular contraction. The chemo-mechanical stiffness modulus *β* has units of stress. This free energy form is introduced to the FEniCS platform^54^ to conduct the finite element simulations in order to predict and optimize the post-contraction mechanical characteristics of the system. The analysis is nonlinear, as material and geometric nonlinearities are considered and also the contact formulation is nonlinear. Linear triangular elements are employed for the simulation in FEniCS.

On a 4 × 4 micropillar substrate, the micropillars are considered rigid and frictionless contact is considered between the cell/fibrin composite and the micropillars. Loading is induced through ramping of the isotropic activation to its maximum value, to mimic the effect of cell contraction on the fibrin network. The micropillar diameter is 400 μm and pillar-to-pillar distance is 200 μm. Young’s modulus of the matrix is taken to be *E* = 1.9 mN μm^−2^ interpolating *E* = 1.9 mN from the results of Ghajaret et al.^55^. Poisson’s ratio is set at $\nu = 0.3$, and the chemo-mechanical stiffness modulus *β* = 2 mN μm^−2^ was calibrated to the experimental results to match the observed in-plane displacements. The shear modulus of matrix is obtained through $\mu = \frac{E}{{2\left( {1 + \nu } \right)}}$ and Lame’s first parameter through $\lambda = \frac{{E\nu }}{{\left( {1 + \nu } \right)\left( {1 - 2\nu } \right)}}$. The simulations are under plane-stress conditions, consistent with the calibration procedure to obtain the model parameters. A simulation is performed starting from zero activation, ramping up to full activation to study the effect of cell contraction, leading to large deformations to the microtissue as observed in the simulations and in agreement with experimental observations. The units of stress in the results are in mN μm^−2^ and displacement units are μm. The strains for this large deformation problem are quantified through the Euler–Lagrange strain tensor defined as $E_{IJ} = F_{iI}F_{jJ} - I_{IJ}$, where $I$ is the unit second-order tensor.

### Transfer of microvascular meshes

Owing to intrinsic elasticity and resilience, microvascular mesh on micropillar substrate can be manipulated and transferred to different substrates. An intact microvascular mesh at centimeter scale could float after immersing the micropillar substrate in phosphate-buffered saline (PBS) solution. When a rod was placed beneath the floating microvascular mesh and gently lifted up, the microvascular mesh wraps onto the rod.

Alternatively, to transfer a microvascular mesh onto different substrates, a PDMS frame (pre-soaked in 50 μg mL^−1^ fibronectin solution for 1 h at 37 °C to promote cell adhesion) or glass cylinder was placed on top of the micropillar substrate. During overnight culture, while the cells between micropillars organized into a mesh, the PDMS frame or glass cylinder provided cell adhesive points over the entire mesh. The microvascular mesh can then be easily transferred by gently peeling the frame/cylinder off of the micropillar substrate.

### Vascularization of diffusion chamber by microvascular meshes

Circular PDMS frames (inner diameter: 5 mm, outer diameter: 6 mm, thickness: 0.6 mm) were cut from a PDMS membrane using biopsy punches (diameters: 5 and 6 mm, Sklar). Diffusion chamber without islets were fabricated by attaching a circular nylon grid (diameter: 6 mm, pore size: 70 μm, Component Supply) to either side of the PDMS frame. PDMS pre-polymer solution was used as glue. After being coated with 1% (w/v) Pluronic^®^ F127 (Sigma) solution overnight, the void space in chamber device was filled with fibrin matrix (18 mg mL^−1^) to minimize the cell infiltration and tissue ingrowth into the chamber in vivo.

In all in vivo experiments, microvascular mesh was made of ECs and NHDFs (ECs:NHDFs=9:1). NHDFs were added to enhance vascularization and maturation of vasculatures. As previously described, microvascular mesh was assembled in the same way on the micropillar substrate. The parameters of a micropillar substrate were as follows: substrate size was 1 × 1 cm, micropillar diameter and height were 400 and 200 μm, respectively, and micropillar-to-micropillar interval was 200 μm. The volume of the interspace between micropillars was approximately 13.6 μL. The total cell number on the micropillar substrate was approximately 1.1 × 10^5^. After microvascular mesh was cultured for 2 days on the micropillar substrate, a fibrin solution (6.0 mg mL^−1^ fibrinogen and 2.0 U mL^−1^ thrombin) was pipetted on top of microvascular mesh and then the chamber was placed in fibrin solution. Another microvascular mesh on a micropillar substrate was flipped and placed in the fibrin solution, on top of the chamber. After 15 min of incubation at 37 °C, microvascular meshes were embedded in fibrin gel and positioned onto the chamber surface, and the void space created by the removal of micropillars was filled with additional fibrin gel (Mesh device, Supplementary Fig. 11). The Mesh device was cultured in EGM-2 medium for approximately 4 h prior to transplantation. For No cell device, a fibrin solution (6.0 mg mL^−1^ fibrinogen and 2.0 U mL^−1^ thrombin) without cells was gelled around the chamber and cultured for 2 days prior to transplantation. For the Random device, a fibrin solution (same volume used for the Mesh device) containing a random mixture of ECs and NHDFs (same cell amount used for making the microvascular mesh) was placed on top of the chamber and gelled at 37 °C. And then the bottom of the chamber was placed with cells in the fibrin matrix in the same way. The Random device was cultured for 2 days prior to transplantation.

SCID-Beige mice (male and female, model number CBSCBG, 6–8 weeks old, Taconic Biosciences) were anesthetized using 2.5% (v/v) isofluorane in oxygen and maintained at the same rate throughout the procedure. Three ~2.5 cm^2^ squares were shaved (two on one flank and one on the opposite flank of the mouse) and sterilized with alternating scrubs of betadine and 70% (v/v) ethanol. A small ~0.8 cm incision was created using scissors and a subcutaneous pocket was created through blunt dissection. This process was repeated on the opposite flank. The Mesh device was placed in subcutaneous pocket and sutured using 5–0 nylon. For controls, No cell and Random devices were also implanted.

### Isolation and introduction of rat islets into devices

All animal procedures received ethical approval from the Institutional Animal Care and Use Committee at the Cornell University. Mouse and rat experiments have complied with all relevant ethical regulations for animal testing and research.

Sprague-Dawley rats (male, strain code 400, 250–275 g weight, Charles River) were used for harvesting islets. The bile duct was cannulated and the pancreas was distended by injection of 0.15% Liberase (Research Grade, Roche) in RPMI 1640 medium (Gibco). The pancreas was digested at 37 °C for 30 min. Digested pancreases were washed, filtered through a 450-μm sieve, and then suspended in a Histopaque 1077 (Sigma)/M199 medium (Gibco) gradient and centrifuged at 1700 × *g* at 4 °C. This gradient centrifugation step was repeated for obtaining purified islets. The islets were collected from the gradient and further isolated by a series of gravity sedimentations, in which each supernatant was discarded after 4 min of settling. Purified islets were cultured in RPMI 1640 medium overnight for further use. GFP-expressing islets were isolated from SD-Tg(UBC-EGFP)2BalRrrc rats (or SD-EGFP, male, 8–12 weeks old, Rat Resource and Research Center). Approximately 500–1000 islets can be isolated from each rat.

To load rat islets into the diffusion chamber, a fibrin solution (6.0 mg mL^−1^ fibrinogen and 2.0 U mL^−1^ thrombin) containing rat islets (approximately 500 IEQ) was introduced into an 8 mm circular PDMS frame (inner diameter: 7 mm, thickness: 0.6 mm) with nylon grid at the bottom. A second nylon grid was placed on top of the PDMS frame and glued by gelling fibrin at 37 °C for 15 min. And then a fibrin solution (6.0 mg mL^−1^ fibrinogen and 2.0 U mL^−1^ thrombin) was pipetted on top of a HUVEC microvascular mesh on a micropillar substrate, which had been cultured for 2 days in EGM-2 medium. The chamber containing rat islets was placed in the fibrin solution, and another HUVEC microvascular mesh on a micropillar substrate was flipped and placed in the fibrin solution, on top of the chamber. After 15 min of incubation at 37 °C, HUVEC microvascular meshes attached on both sides of the chamber and the void space created by the removal of micropillars was filled with additional fibrin gel (Mesh device, *n* = 14, from 4 isolation and encapsulation experiments, Supplementary Fig. 14). For No cell device (*n* = 9, from 3 isolation and encapsulation experiments), a fibrin solution containing rat islets was loaded into a PDMS frame with a nylon grid at the bottom. Another nylon grid was placed on top of the PDMS frame and glued by gelling fibrin at 37 °C for 15 min. The Random device (*n* = 11, from 3 isolation and encapsulation experiments) was prepared in the same way as No cell device except the random mixture of ECs and NHDFs in fibrin matrix had been cultured on the nylon grids for 2 days. Subcutaneous transplantation was performed as previously described for the empty device without islets. In iPSC-EC and rat islet transplantation, *n* = 5 in No iPSC-EC group, *n* = 6 in Random iPSC-EC group, and *n* = 6 in Mesh iPSC-EC group were from 2 isolation and encapsulation experiments.

### Evaluation of hyperglycemia correction in diabetic mice

To create insulin-dependent diabetic mice, healthy SCID-Beige mice were treated with freshly prepared STZ solution (120 mg kg^−1^ mouse) twice via intraperitoneal injection. BG level of all the mice was retested prior to transplantation. Only mice whose non-fasting BG level was >350 mg dL^−1^ were considered diabetic. Mice were randomized among the control and treatment groups to keep BG levels at the similar level for all groups at the beginning of transplantation.

Non-fasting BG levels were monitored twice during the first week and then once a week following the transplant surgery. BG measurements were randomly performed by blinded or non-blinded personnel. A small droplet of blood was collected from tail vein and glucose concentration was measured with a commercial glucometer (Clarity Plus, Diagnostic Test Group). Mice with non-fasting BG levels <200 mg dL^−1^ were considered normoglycemic. After retrieving the devices from diabetic mice, non-fasting BG was monitored for one more week. The retrieved devices were fixed in 10% formalin, embedded in paraffin, and sectioned. H&E staining and immunostaining of the sections were performed.

IPGTT was measured 1 month and 3 months after surgery. The mice were fasted for approximately 16 h. Glucose was administered via intraperitoneal injection (2.0 g kg^−1^ mouse), and BG levels were measured at the indicated times.

### Mouse blood vessel perfusion

To image anastomoses between mouse and human blood vessels, two types of lectins (100 μL each) were injected into mouse through tail vein after 14 days of transplantation. Green fluorescein UEA-I lectin (2 mg mL^−1^) specifically bound to human endothelial cells^6^. Red DyLight 594-labeled GSL-I isolectin B_4_ (1 mg mL^−1^) specifically bound to mouse endothelial cells^27^. Mice were euthanized, and devices were harvested and fixed in 10% (v/v) formalin. Blood vessels were imaged with a laser scanning confocal microscope (LSCM, FLUOVIEW FV1000, Olympus). To evaluate the earliest time point that blood-perfused human vasculatures were formed and anastomosed with mouse vascular system, microvascular meshes of HUVEC-GFP/NHDF (9:1, Mesh device) and random mixture of HUVEC-GFP/NHDF (9:1, Random device) were subcutaneously implanted, and mice were perfused with lipophilic carbocyanine dye DiI^28^ at different time points (Days 4, 7, and 10). Briefly, a butterfly needle was inserted into left ventricle of the mouse, and 2 mL of PBS, 10 mL of DiI solution, and 10 mL of 4% (v/v) paraformaldehyde solution were perfused sequentially at the speed of 2 mL min^−1^ using a syringe pump. Immediately after perfusion, devices were retrieved and imaged using laser scanning confocal microscope. The overlap of green HUVEC-GFP and red dye DiI indicated that human vasculatures were blood-perfused and connected with mouse vascular system. To visualize the vascularization of implanted rat islets, mouse blood vessels were perfused with dye DiI and retrieved devices were imaged in the same way.

### Immunohistochemistry and image analysis

Microvascular meshes cultured for 2 days in vitro were fixed in 10% formalin, permeabilized with 0.2% (v/v) Triton X-100, and blocked with 2% (w/v) bovine serum albumin solution. Human CD31 was detected with a primary mouse anti-human CD31 antibody (1:200 dilution, eBioscience, Cat. No. 14–0319–82) followed by a secondary Alexa Fluor^TM^ 488 goat anti-mouse antibody (1:400 dilution, Invitrogen, Cat. No. A-11001) or Alexa Fluor^TM^ 555 donkey anti-mouse antibody (1:400 dilution, Invitrogen, Cat. No. A-31570). F-actin was stained with Texas-Red^TM^-X (1:40 dilution, Invitrogen, Cat. No. T7471) or Alexa Fluor^TM^ 488 phalloidin (1:40 dilution, Invitrogen, Cat. No. A12379). Cell nuclei were counterstained with Hoechst (Invitrogen). The images were obtained using a fluorescence microscope (EVOS, AMG).

Laser scanning confocal microscope (LSCM, FLUOVIEW FV1000, Olympus) was used to image 3D structure of microvascular mesh. Images were acquired using the FV10-ASW2.0 software (Olympus). The cross-sectional images were analyzed with Fiji ImageJ (https://imagej.net/Fiji).

Devices retrieved from mice were fixed in 10% formalin, processed, embedded, and sectioned for immunohistochemistry. The primary antibodies were rabbit anti-human CD31 (1:200 dilution, Sigma, Cat. No. SAB5600061–100UL), goat anti-mouse CD31 (1:200 dilution, R&D systems, Cat. No. AF3628), and α-SMA antibody conjugated with Cy3 (1:200 dilution, Sigma, Cat. No. C6198-.2 ML). The secondary antibodies were Alexa Fluor^TM^ 594 donkey anti-rabbit antibody (1:400 dilution, Invitrogen, Cat. No. R37119), Alexa Fluor^TM^ 488 donkey anti-rabbit antibody (1:400 dilution, Invitrogen, Cat. No. R37118), and Alexa Fluor^TM^ 488 donkey anti-goat antibody (1:400 dilution, Invitrogen, Cat. No. A-11055). After washing with PBS, the slides were mounted in Fluoroshield^TM^ with 4,6-diamidino-2-phenylindole (Sigma) and imaged with a fluorescence microscope (EVOS, AMG).

On H&E staining images, human or mouse blood vessels were identified by luminal structures with erythrocytes inside. The vessel number was quantified by counting individual vessels within the interfacial area between the device and panniculus carnosus muscle. The vessel density (vessels mm^−2^) was calculated by dividing the total vessel number by the interfacial area. The area percentage of vessels was calculated by dividing the total area of erythrocyte-containing luminal structures by the interfacial area. Blood perfusion on Day 10 was quantified by image analysis (Fiji ImageJ (https://imagej.net/Fiji)). The percentage of blood-perfused human vessels was calculated by dividing the pixel area of merged green and red color (human vessels containing perfused dye) with the pixel area of green color (total human vessels). Histological slides of retrieved devices (10 days after transplantation) were stained with human CD31 and α-SMA antibodies. The human vasculatures covered with PVCs were identified by vessel cross-sections that had luminal shape (with erythrocytes inside) and were positively stained by both human CD31 and α-SMA antibodies.

For immunohistochemical detection of rat insulin, histological sections were stained with rabbit anti-rat insulin (1:200 dilution, abcam, Cat. No. ab181547). The secondary antibody was Alexa Fluor^TM^ 594 donkey anti-rabbit antibody (1:400 dilution, Invitrogen, Cat. No. R37119). Cell nuclei were stained with Hoechst (Invitrogen).

### Statistical analysis

Statistical analyses were performed using GraphPad Prism and R. Experiments were repeated at least twice. All mice were used for analysis unless they died or had to be euthanized according to IACUC-approved protocols. Data were analyzed using unpaired two-tailed *t* test (Fig. 3i and Supplementary Fig. 13b), one-way analysis of variance (Figs. 3d, 4d, and 5e and Supplementary Fig. 18d), or analysis of covariance (ANCOVA) with time treated as continuous covariate (Figs. 4b, c and 6b, c), followed by Tukey post hoc test and 95% confidence interval. All plots represent mean ± standard error of mean. The standard error of mean was illustrated by bars in the figures. *P* values <0.05 were considered to be statistically significant.

### Reporting summary

Further information on research design is available in the Nature Research Reporting Summary linked to this article.

## Supplementary information

Supplementary Information

Description of Additional Supplementary Files

Supplementary Movie 1

Supplementary Movie 2

Supplementary Movie 3

Supplementary Movie 4

Reporting Summary

## Data Availability

All relevant data are available within the article and Supplementary Information and from the corresponding author upon reasonable request.
