# Supplementary material for: Engineering transferrable microvascular meshes for subcutaneous islet transplantation
Source: Nat Commun. 2019 Oct 10;10:4602. doi: 10.1038/s41467-019-12373-5 (PMC6787187; doi:10.1038/s41467-019-12373-5)
Supplement: Supplementary file 1 — Supplementary Information [file 41467_2019_12373_MOESM1_ESM.pdf]

Engineering transferrable microvascular meshes for subcutaneous islet transplantation

Song et al.

Supplementary information

Engineering transferrable microvascular meshes for subcutaneous islet transplantation

Wei Song[1], Alan Chiu[1], Long-Hai Wang[1], Robert E. Schwartz[2], Bin Li[3], Nikolaos Bouklas[3], Daniel T. Bowers[1], Duo An[1], Soon Hon Cheong[4], James A. Flanders[4], Yehudah Pardo[5], Qingsheng Liu[1], Xi Wang[1], Vivian K. Lee[6], Guohao Dai[6], and Minglin Ma[1, *]

[1] Department of Biological and Environmental Engineering, Cornell University, Ithaca, NY 14853, USA.

[2] Division of Gastroenterology & Hepatology, Department of Medicine, Weill Cornell Medical College, New York, NY 10021, USA.

[3] Department of Mechanical and Aerospace Engineering, Cornell University, Ithaca, NY 14853, USA.

[4] Department of Clinical Sciences, Cornell University, Ithaca, NY 14853, USA.

[5] Nancy E. and Peter C. Meinig School of Biomedical Engineering, Cornell University, Ithaca, NY 14853, USA.

[6] Department of Bioengineering, Northeastern University, Boston, MA 02120, USA.

*Correspondence and requests for materials should be addressed to M.M. (email: mm826@cornell.edu)

Supplementary Tables

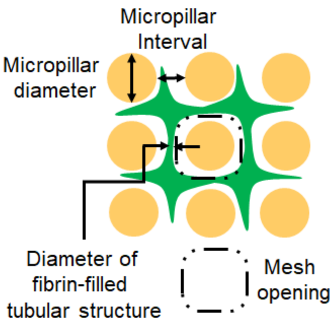

Micropiller Diameter-Interval (μm)	Diameter of Fibrin-Filled Tubular Structure (μm)	Mesh Opening Size (μm)	Mesh Opening Density (openings mm^{-2})
100-50	15.0 ± 0.5 (n=75)	134.9 ± 0.5 (n=75)	44
200-50	36.9 ± 0.5 (n=185)	213.1 ± 0.5 (n=185)	16
400-50	23.1 ± 0.6 (n=49)	427.0 ± 0.6 (n=49)	5
400-100	51.6 ± 1.8 (n=48)	448.4 ± 1.8 (n=48)	4
400-200	133.0 ± 1.5 (n=131)	467.0 ± 1.5 (n=131)	2

Supplementary Table 1. Characterization of the diameter of fibrin-filled tubular structure, and the size and density of mesh opening on different micropillar substrates. Micropillar is yellow and fibrin-filled tubular structure is green. The density of mesh opening is equal to the number of micropillars in 1 mm^2 area.

Transplantation days	No cell (normoglycemic mice/total mice)	Random (normoglycemic mice/total mice)	Mesh (normoglycemic mice/total mice)
4	0/9	2/11	4/14
7	0/9	2/11	5/14
14	0/9	2/11	10/14
21	1/9	5/11	8/14
28	0/9	3/11	10/14
35	1/9	1/11	10/14
42	0/9 (Devices retrieved from 9 mice)	1/11 (Devices retrieved from 10 mice)	8/14 (Devices retrieved from 11 mice)
49		1/1	3/3
56		1/1	3/3
63		1/1	3/3
70		0/1	3/3
77		0/1	3/3
84		1/1	3/3
91		0/1 (Device retrieved)	3/3 (Devices retrieved from 2 mice)
98			1/1
105			1/1
112			0/1 (Device retrieved)

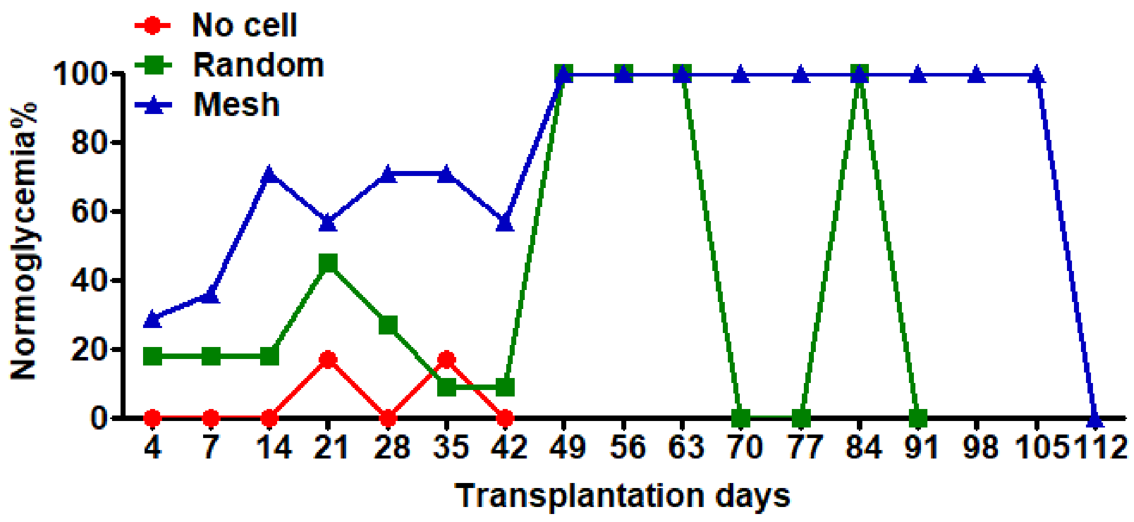

Supplementary Table 2. The summary of the number and percentage of normoglycemic mice (non-fasting blood glucose<200 mg dL^{-1}) in No cell, Random, and Mesh devices during 112 days of transplantation. (Note that the fluctuations in the %normoglycemia graph were likely due to the food intake/measurement variation and the small sample number at later time points.)

Transplantation days	No iPSC-EC (minormoglycemic mice/total mice)	Random iPSC-EC (mnormoglycemic mice/total mice)	Mesh iPSC-EC (normoglycemic mice/total mice)
4	0/5	1/6	1/6
7	0/5	0/6	1/6
14	0/5 (One sick mouse euthanized)	0/6	4/6
21	0/4	1/6	2/6
28	0/4	1/6	3/6
35	0/4	1/6	3/6
42	0/4 (One sick mouse euthanized)	1/6	3/6
49	0/3	1/6	2/6
56	0/3	1/6 (Two sick mice euthanized)	2/6
63	0/3	1/4	3/6
70	0/3	1/4	3/6
77	0/3	0/4	3/6
84	0/3	0/4	2/6
91	0/3 (Device retrieved)	0/4 (Device retrieved)	2/6 (Device retrieved)

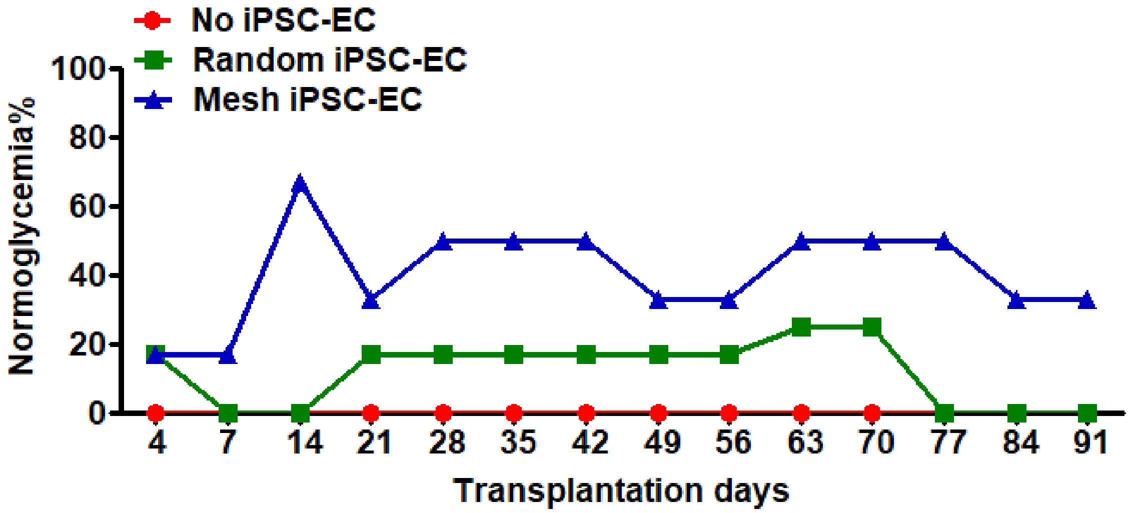

Supplementary Table 3. The summary of the number and percentage of normoglycemic mice (non-fasting blood glucose<200 mg dL^{-1}) in No iPSC-EC, Random iPSC-EC, and Mesh iPSC-EC devices during 91 days of transplantation. (Note that the fluctuations in the %normoglycemia graph were likely due to the food intake/measurement variation and the small sample number at later time points.)

Supplementary Figures

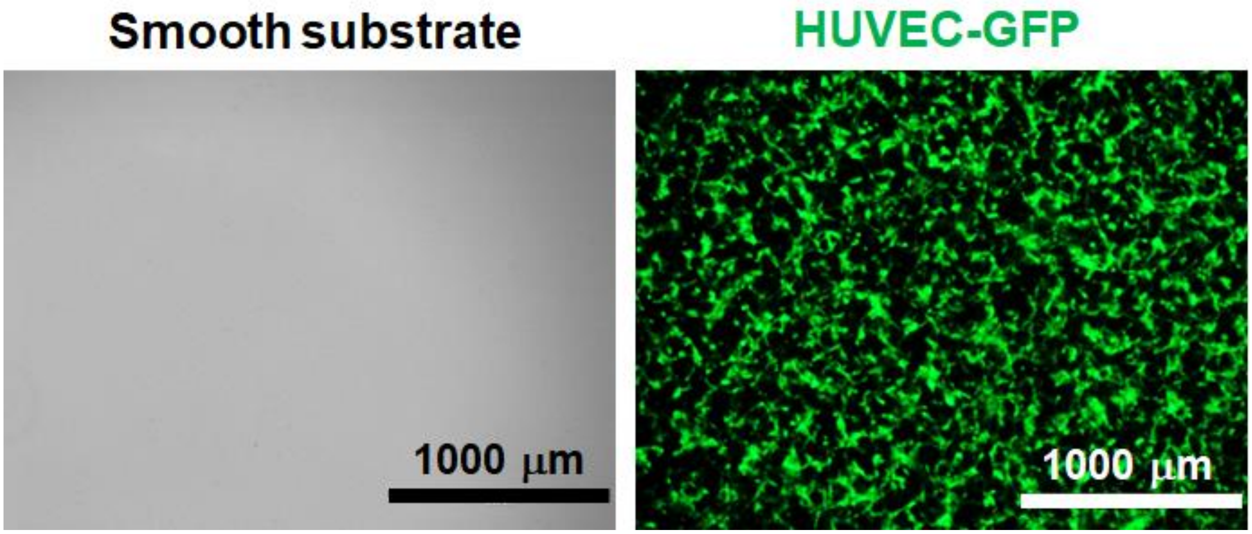

Supplementary Figure 1. The organization of HUVECs (expressing GFP) is random in a fibrin matrix on a smooth substrate without micropillars after 2 days of culture.

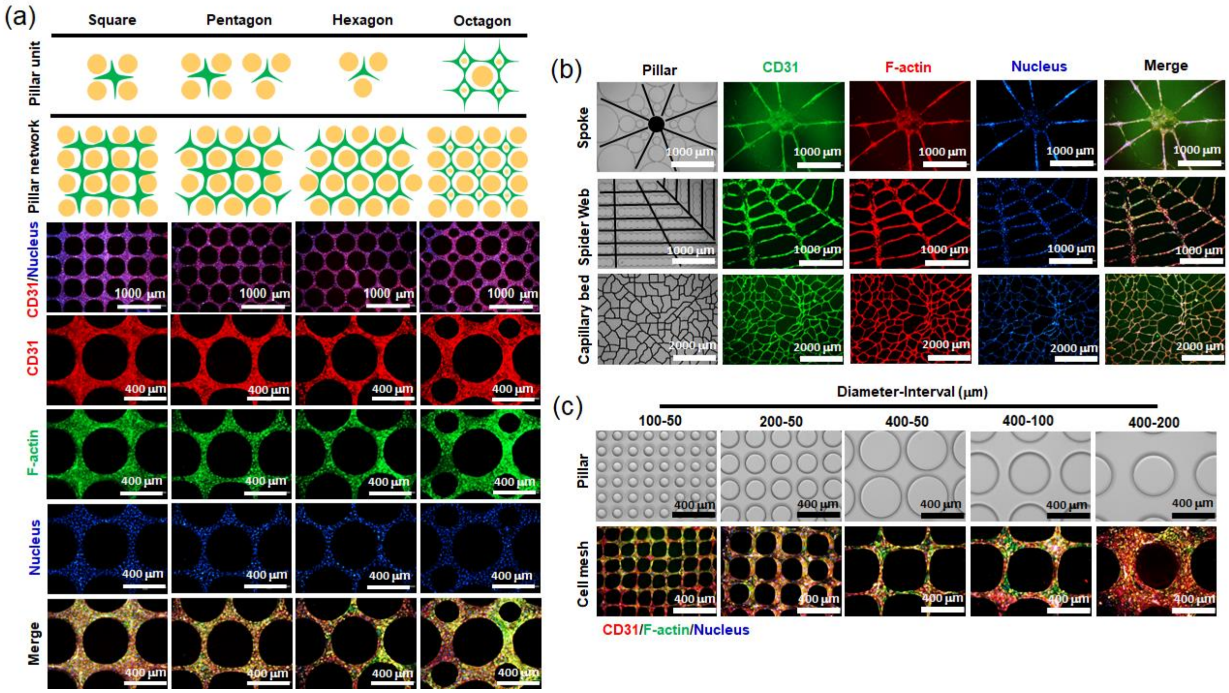

Supplementary Figure 2. ASA-enabled HUVEC microvascular meshes with different geometries and dimensions. (a) The arrangement and combination of micropillars with different sizes control organization of HUVECs into square, pentagon, hexagon, and octagon shapes. (b) More complex geometries are realized by changing micropillar arrangement. (c) The micropillar diameters and micropillar-to-micropillar intervals modulate the diameter of fibrin-filled tubular structure, and the size and density of microvascular mesh openings.

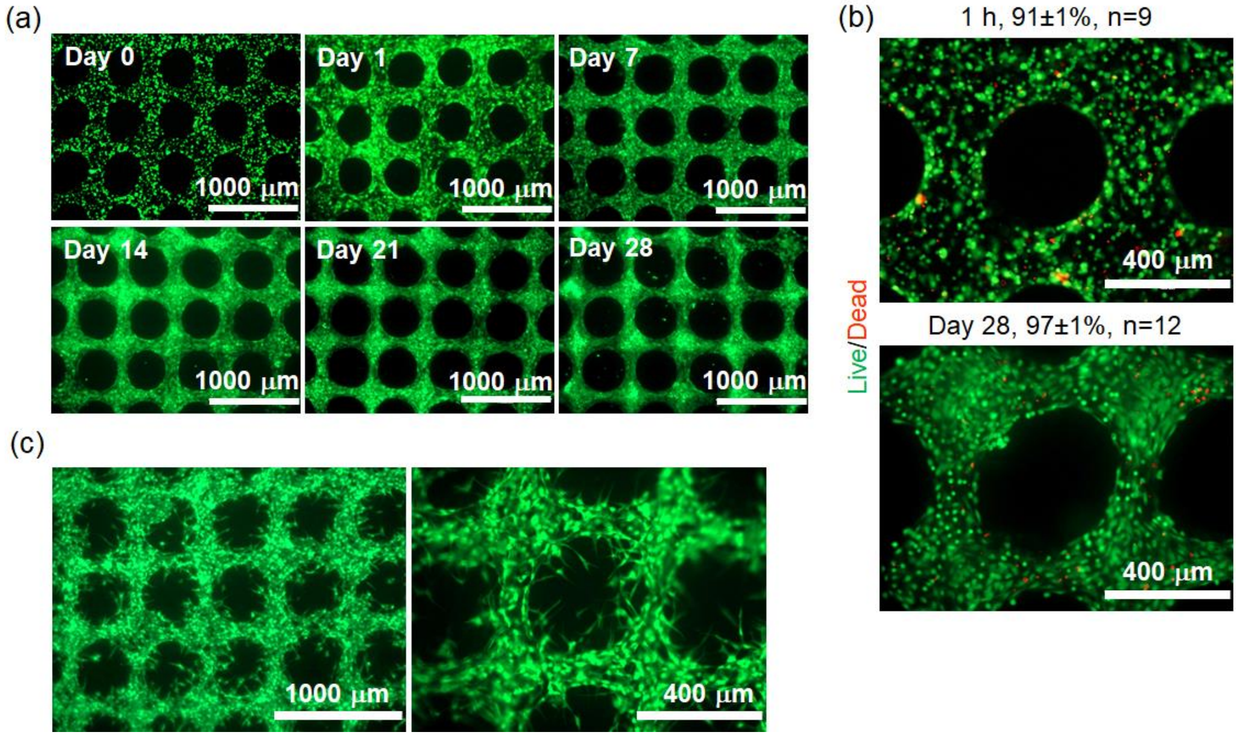

Supplementary Figure 3. (a) The HUVEC (expressing GFP) microvascular mesh maintains square shape for 28 days on a micropillar substrate. (b) The live (green)/dead (red) staining indicates a high cell viability in microvascular mesh after 1 hour and 28 days of culture on the micropillar substrate. The quantified cell viability is 91±1% (n=9) after 1 hour of culture and 97±1% (n=12) after 28 days of culture. (c) The iPSC-EC (expressing GFP) microvascular mesh generates numerous angiogenic sprouts after embedded in a fibrin matrix after 2 weeks of culture.

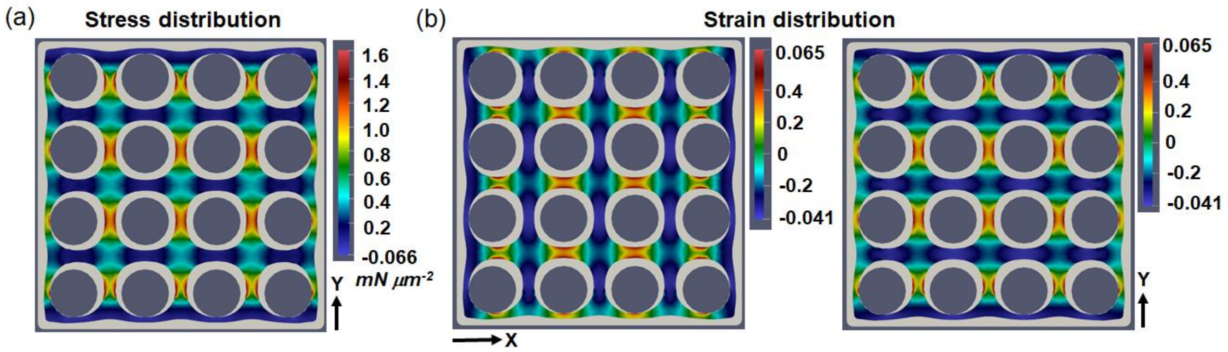

Supplementary Figure 4. The contraction simulation shows the normal stress distribution in the Y (Cauchy stress component σ_{22}) direction (a) and normal strain distribution $E_{11}$and E_{22} in the X and Y directions (b) on a 4×4 micropillar substrate (dark gray). The initial shape of cells and fibrin matrix is displayed in light gray. The unit of stress is mN μm^{-2}. The micropillar diameter is 400 μm and micropillar-to-micropillar distance is 200 μm.

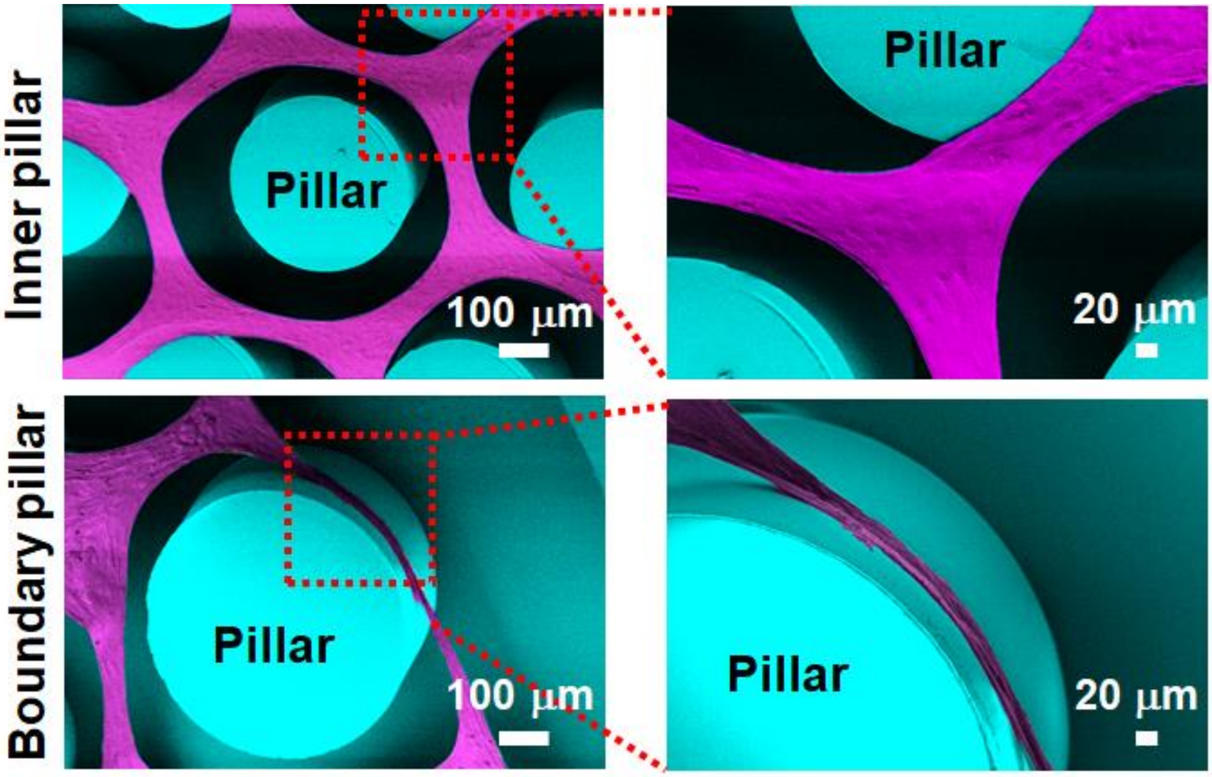

Supplementary Figure 5. SEM images of a HUVEC hexagon mesh at the inner and boundary regions on a micropillar substrate. The cellular structures wrap around micropillars at the boundary to support entire mesh and prevent its shrinkage. The micropillars are pseudo-colored as blue and HUVECs are pseudo-colored as purple.

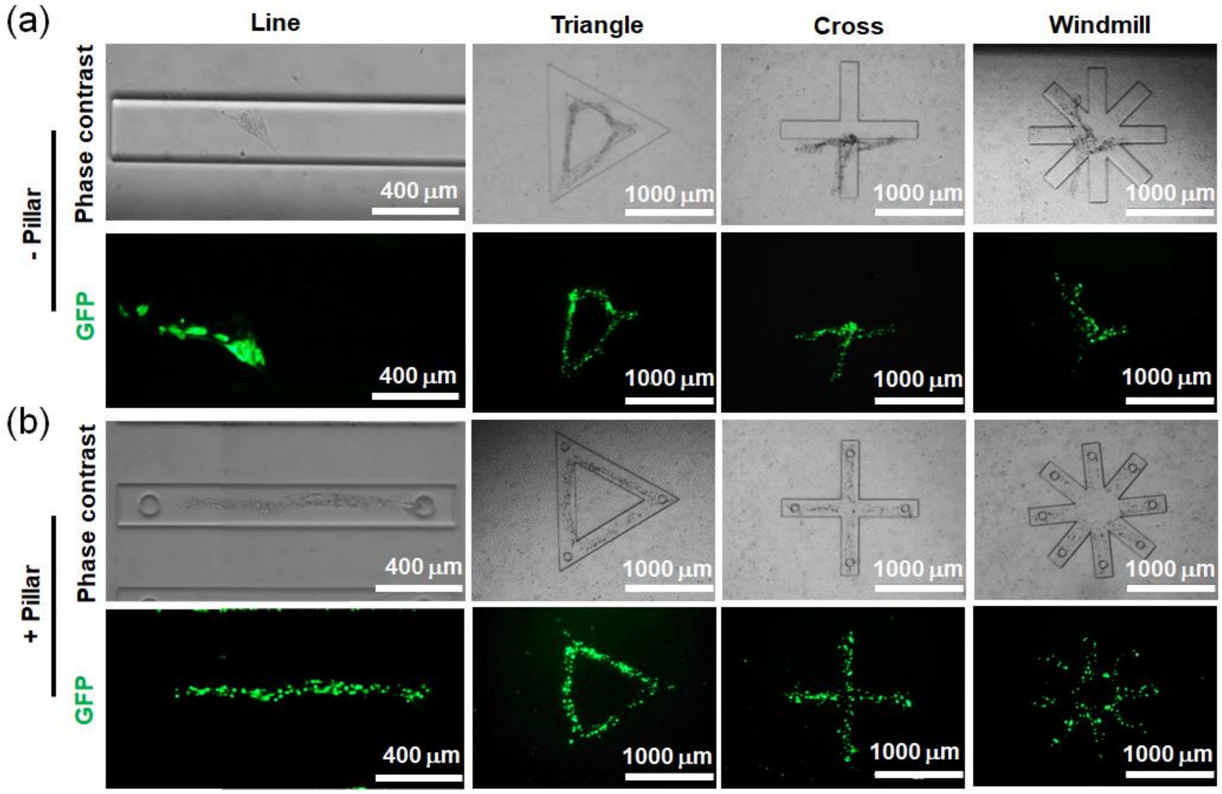

Supplementary Figure 6. (a) HUVECs (expressing GFP) in a fibrin matrix shrink into random clumps in different geometric grooves without micropillars inside. (b) HUVECs in a fibrin matrix organize into corresponding shapes in different geometric grooves with micropillars inside.

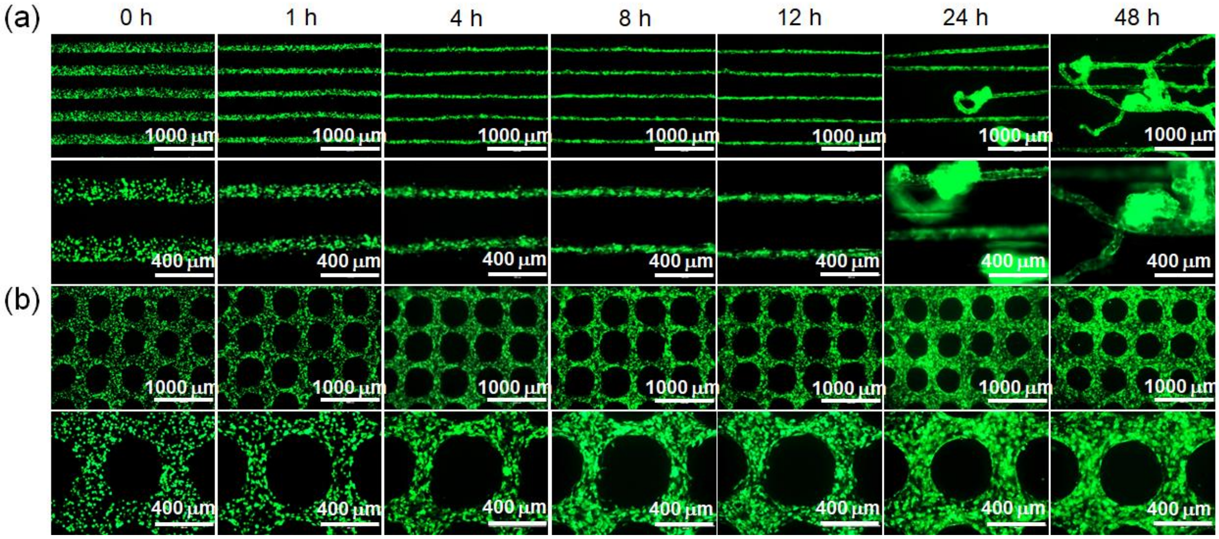

Supplementary Figure 7. HUVECs (expressing GFP) in fibrin matrix gradually shrink into clumps in groove without micropillars inside after 48 hours of culture (a); in contrast, HUVEC microvascular meshes are stable on a micropillar substrate (b).

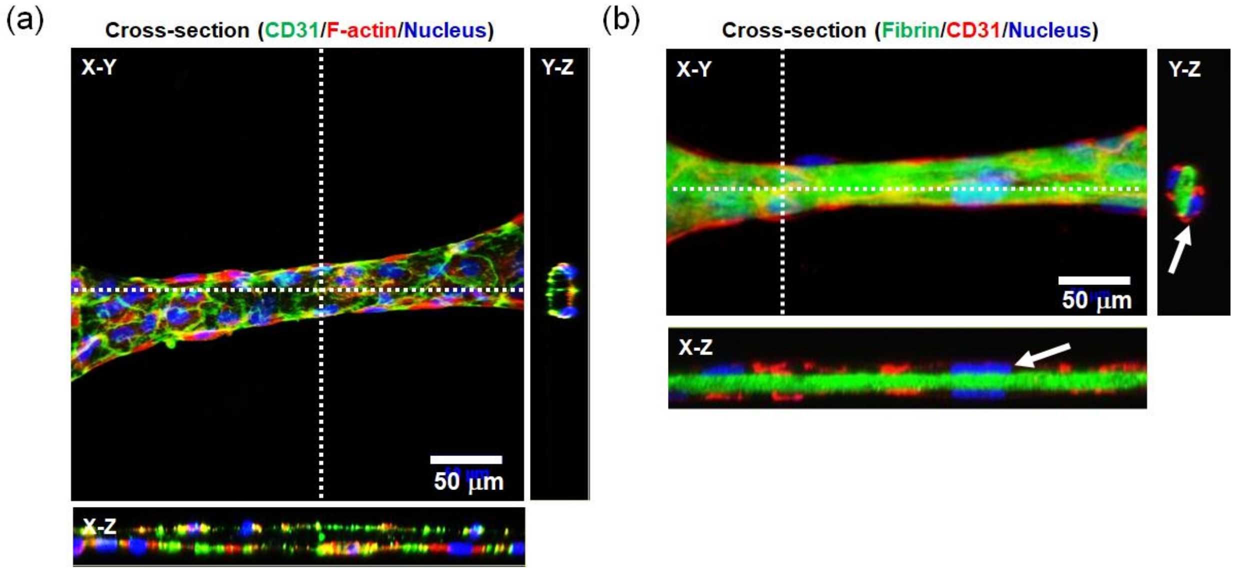

Supplementary Figure 8. (a) Cross-sectional confocal image of a HUVEC mesh (contracted region) organized on a micropillar substrate after 14 days of culture. Human CD31 antibody is green, F-actin is red, and nucleus is blue. (b) Cross-sectional confocal image of HUVECs attached around a fibrin matrix. The fibrin matrix is green, human CD31 antibody is red, and nucleus is blue. White arrow points to HUVECs attached on fibrin matrix.

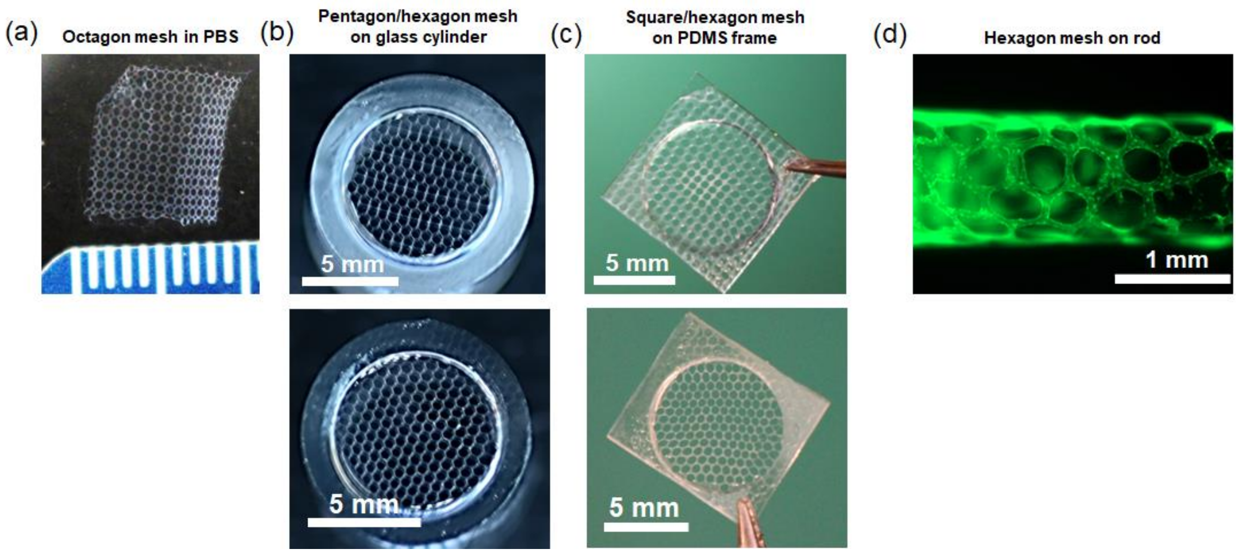

Supplementary Figure 9. Transfer of microvascular meshes to different substrates. (a) An octagon mesh of HUVECs floating in PBS. (b) Pentagon/hexagon meshes of HUVECs transferred to glass cylinders. (c) Square/hexagon meshes of HUVECs transferred to PDMS frames. (d) A hexagon mesh of human iPSC-ECs transferred to a rod.

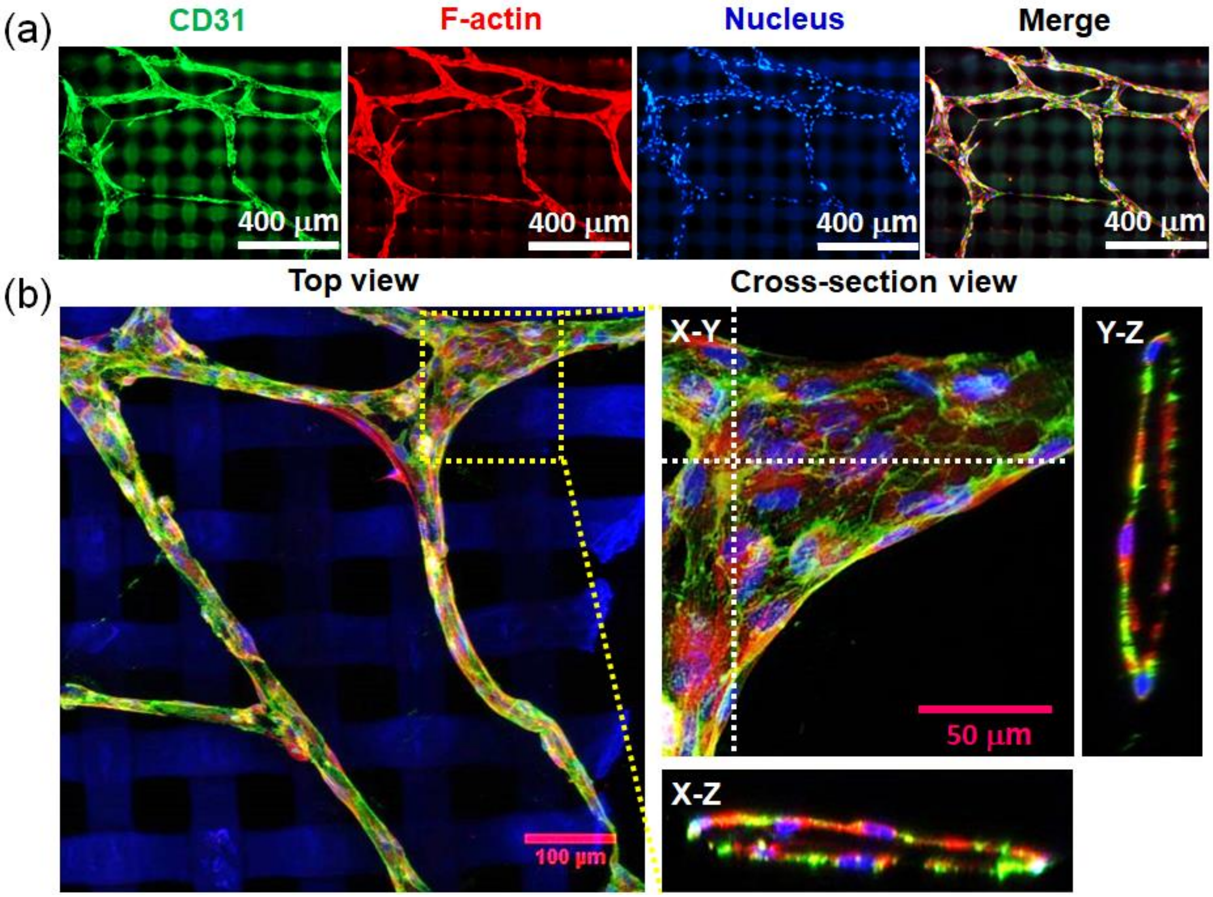

Supplementary Figure 10. Immunostaining images of a HUVEC capillary-like mesh on top of a device. (a) The fluorescent images of stained human CD31 antibody (green), F-actin (red), and nucleus (blue). Nylon grid on the device has auto-fluorescent color. (b) Top-view and cross-sectional view of the HUVEC mesh. Capillary mesh transferred to the device maintained integrity and luminal structure.

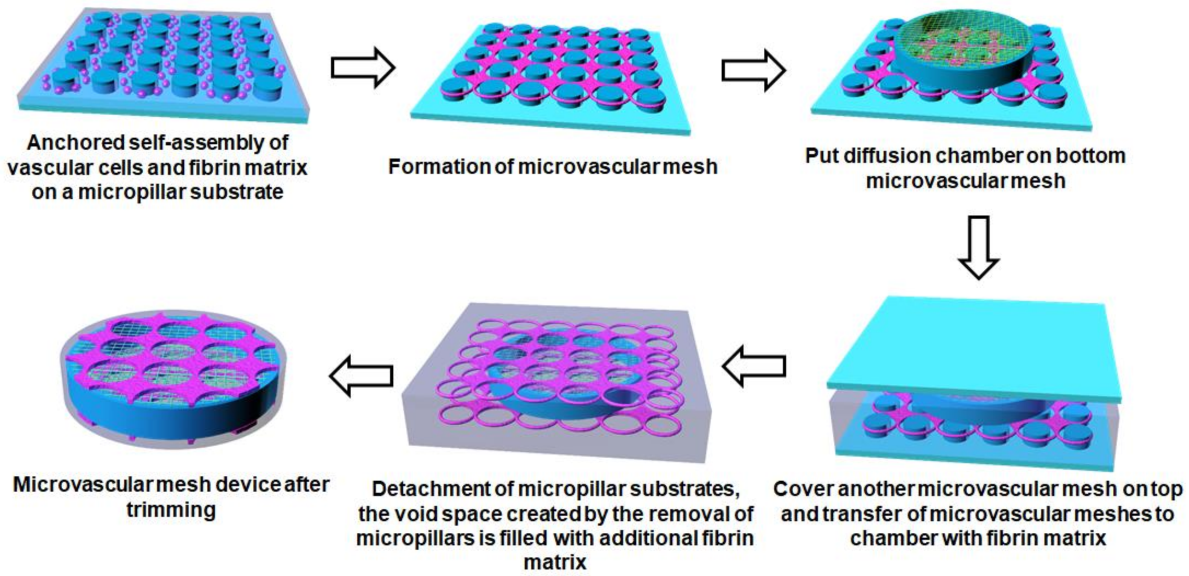

Supplementary Figure 11. Schematic illustration of the fabrication process of microvascular mesh device. First, vascular cell suspension in fibrin matrix is placed on a micropillar substrate. Second, microvascular mesh is formed via anchored self-assembly on a micropillar substrate. Third, to transfer microvascular mesh, a diffusion chamber device is placed on top of a microvascular mesh. Forth, a fibrin matrix is added and another microvascular mesh is placed on the device; After 15 min of incubation at 37 °C, microvascular meshes are embedded in fibrin matrix and attached to the device. Fifth, two micropillar substrates are removed and the void space created by the removal of micropillars is filled with additional fibrin matrix. Lastly, microvascular meshes and fibrin matrix are trimmed to the size of the device.

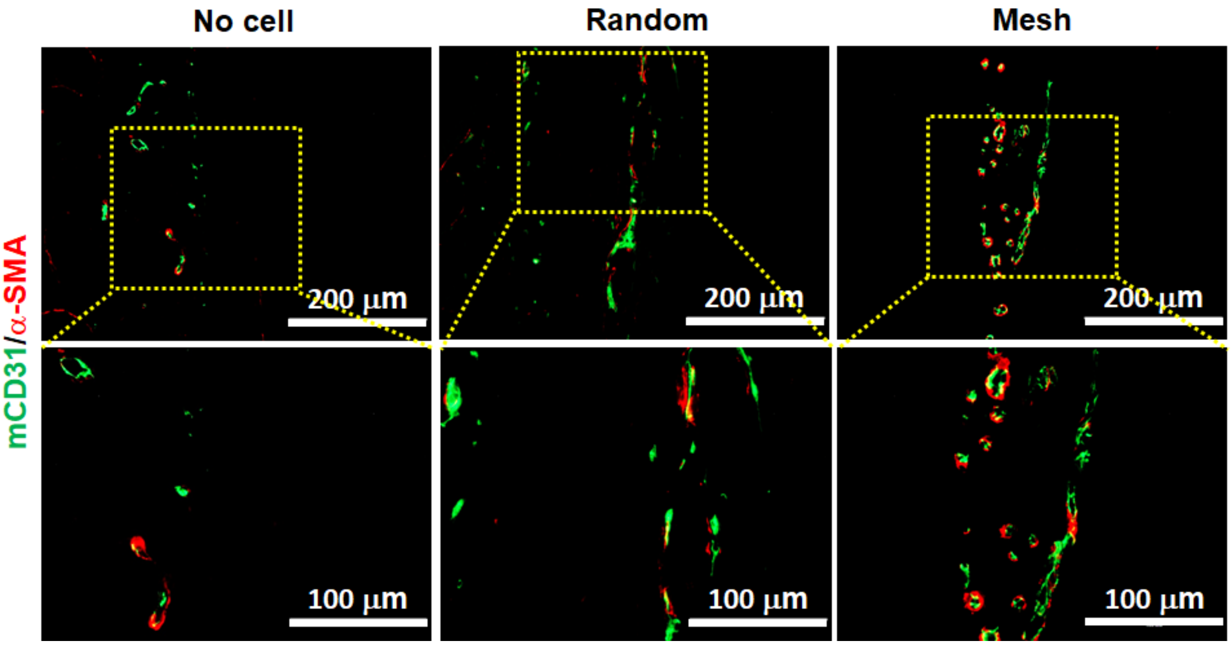

Supplementary Figure 12. Immunostaining images of blood vessels covered by perivascular cells at the interface between the device and panniculus carnosus muscle after 2 weeks of subcutaneous transplantation. Mouse CD31 antibody is green and α-smooth muscle actin (α-SMA) is red.

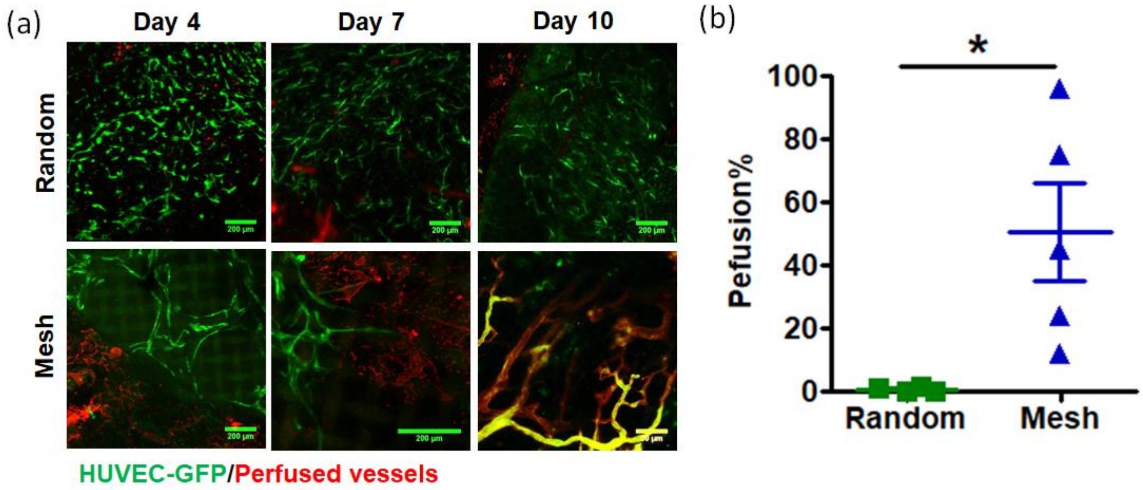

Supplementary Figure 13. (a) The gradual formation of blood-perfused human vasculatures which are anastomosed with mouse vascular system during 10 days post transplantation in Mesh device. On day 4 and 7, perfused mouse vasculatures are found nearby the microvascular meshes of HUVEC-GFP, however, the blood-perfused human vasculatures have not been generated and connected to mouse vascular system. On day 10, human vasculatures derived from microvascular mesh are functional and connected to mouse vascular system since blood-perfused vessels have overlapped color of green HUVEC-GFP and red dye DiI. It should be noted that original square mesh structures of HUVEC-GFP are gradually re-molded during the vascularization and anastomoses development. In contrast, blood-perfused human vasculatures are not observed in Random device under the confocal microscope on Day 10. (b) The percentage of blood-perfused human vasculatures is 0.7±0.3% (n=4) and 50.4±15.6% (n=5) for Random and Mesh device, respectively, after 10 days post transplantation. * $P<0.05$. Unpaired two-tailed t-test.

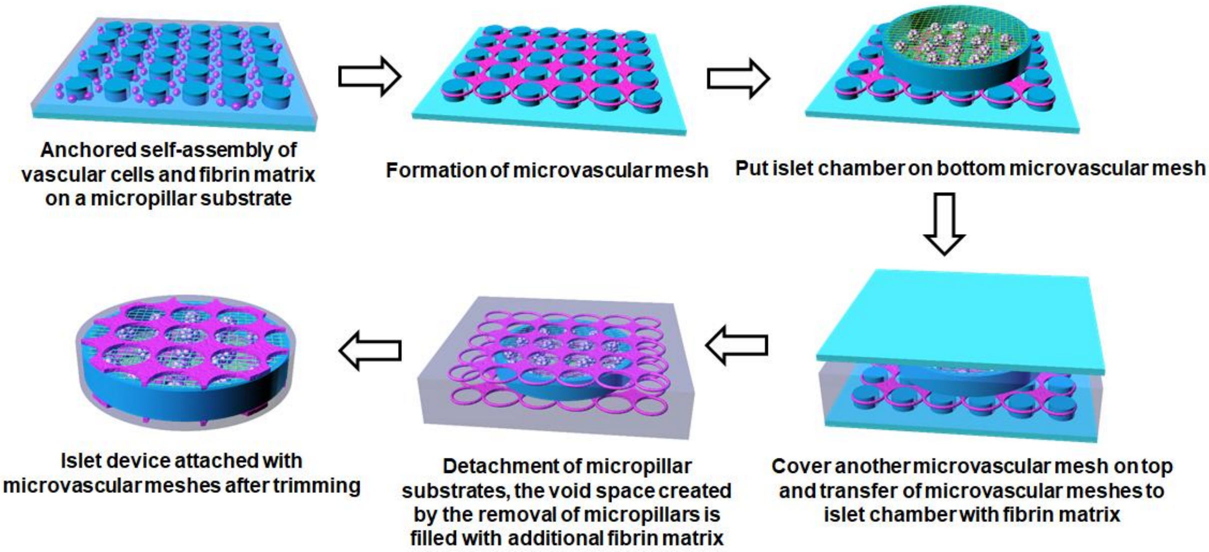

Supplementary Figure 14. Schematic illustration of the fabrication process of microvascular mesh device containing islets. The whole process is similar to that with empty devices (Figure S11) except islets are placed inside the device before microvascular meshes are attached.

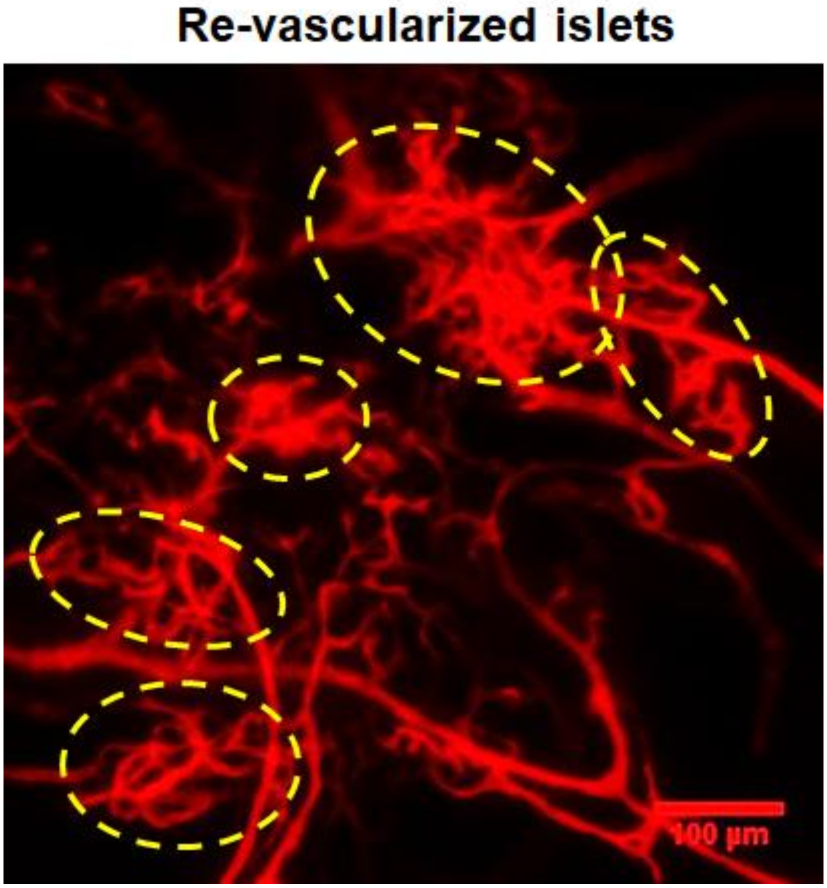

Supplementary Figure 15. Transferrable microvascular mesh attached on the device promotes re-vascularization of rat islets transplanted in the poorly vascularized subcutaneous space of SCID-Beige mice for 6 weeks.

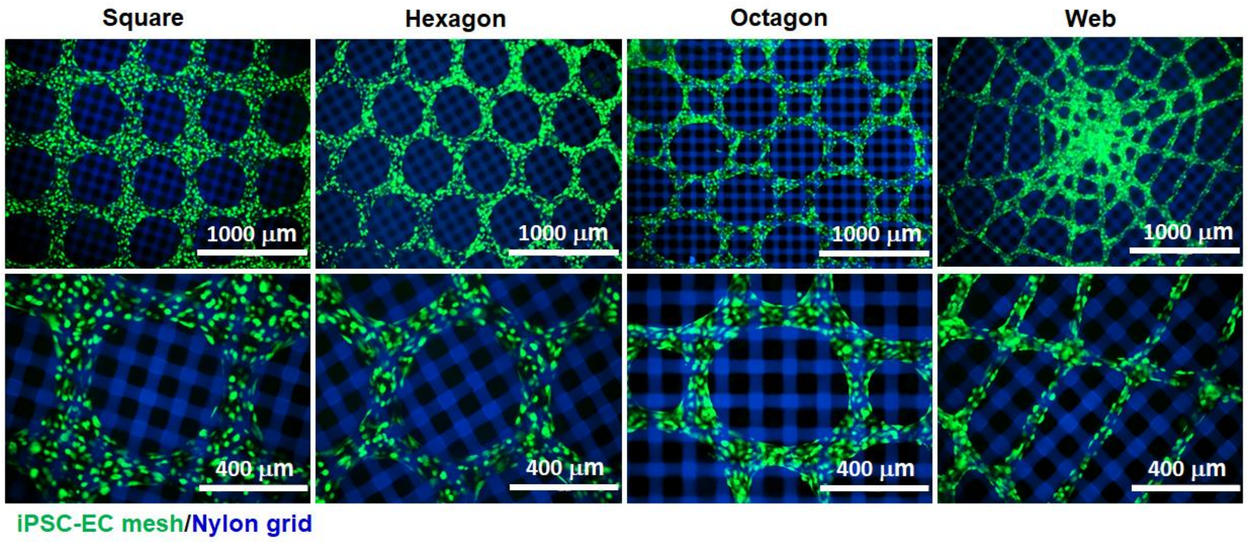

Supplementary Figure 16. Human iPSC-EC meshes with different geometries transferred to devices. Human iPSC-ECs expressing GFP are green and nylon grids on devices are blue.

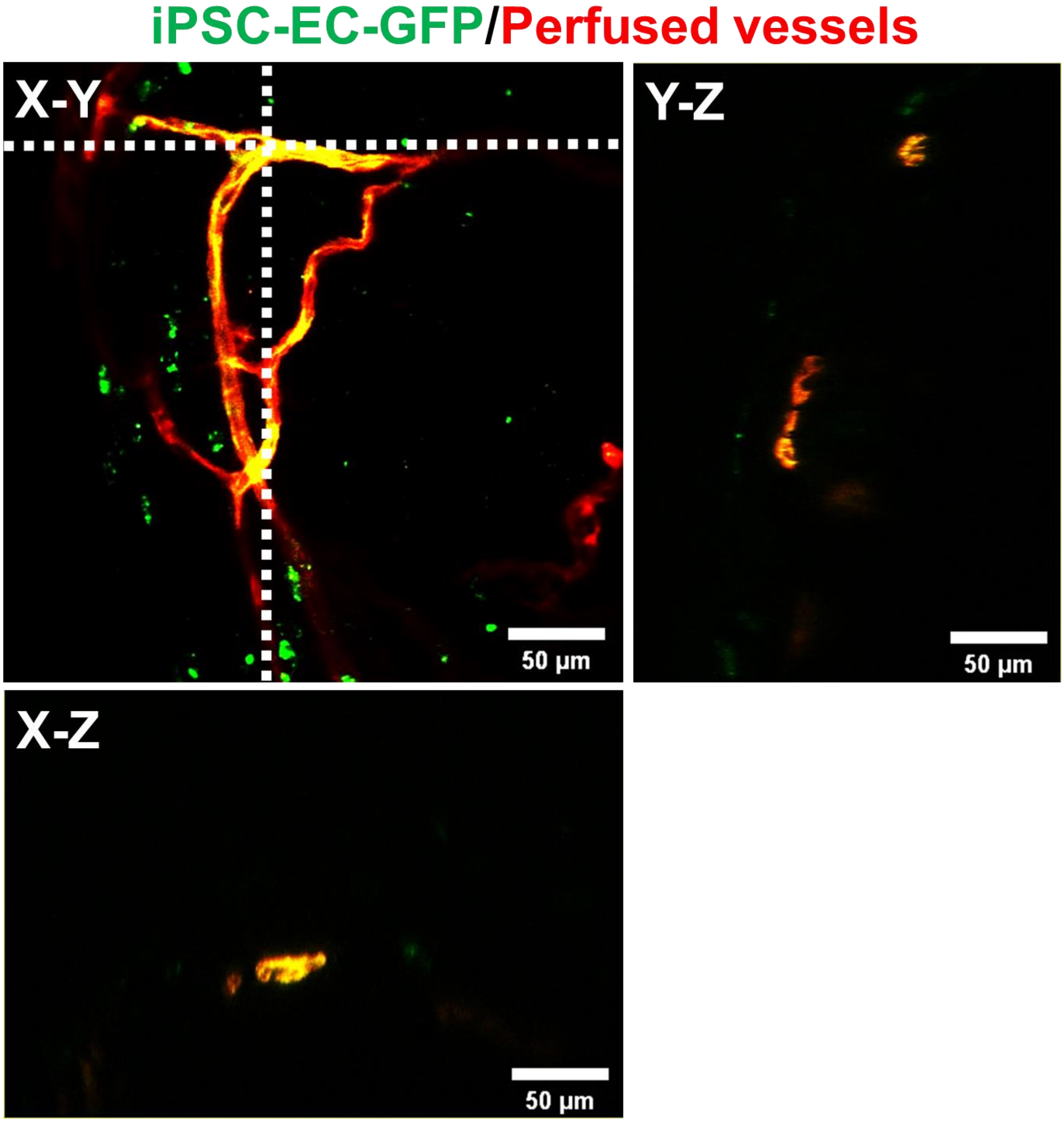

Supplementary Figure 17. The formation of blood-perfused iPSC-EC vasculatures which are anastomosed with mouse vascular system after 10 days post transplantation in iPSC-EC Mesh device. The percentage of blood-perfused iPSC-EC derived human vasculatures is 47.0±20.3% (n=3). iPSC-EC-GFP is green and blood-perfused vessel with dye DiI is red. The overlap of green and red shows yellow.

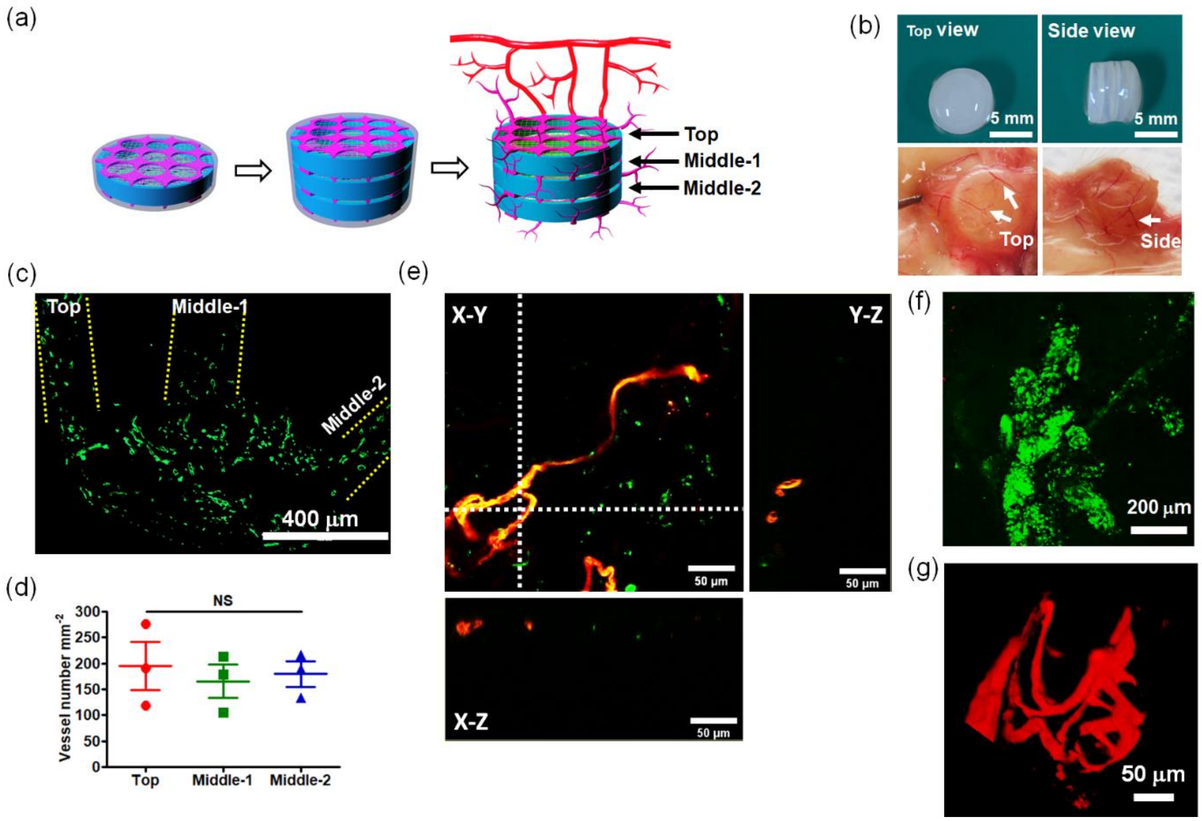

Supplementary Figure 18. A stacked construct consisting of three individual Mesh devices. A high degree of vascularization is observed after two weeks of subcutaneous implantation in SCID-Beige mice. (a) Schematic illustration of the fabrication process of stacked construct. Three individual “Mesh” devices are stacked and glued with fibrin matrix, and then subcutaneously transplanted into SCID-Beige mice. (b) Digital photos of the construct prior to implantation and after two weeks of implantation, inside the mice right before retrieval. The arrows point to blood vessels on top and side of the device. The original square network of microvascular mesh was not preserved due to the development and re-modeling of vascularization and anastomoses. (c) Immunostaining image of blood vessels (CD31 antibody, green) on the retrieved construct. The construct was distorted during the histological sectioning but abundant vessels were observed on the top of and between devices. (d) Vessel density at the top layer and middle layers. n=3, NS indicates no significant difference. (e) Confocal image of blood-perfused human vasculatures in the middle layer. HUVEC-GFP is green, perfused dye DiI in vessels is red, and the overlap is yellow. (f) Confocal image of live/dead staining of retrieved rat islets in stacked construct. Live cells are green and dead cells are red. (g) Confocal image of perfused blood vessels in a re-vascularized rat islet.

Supplementary Movies

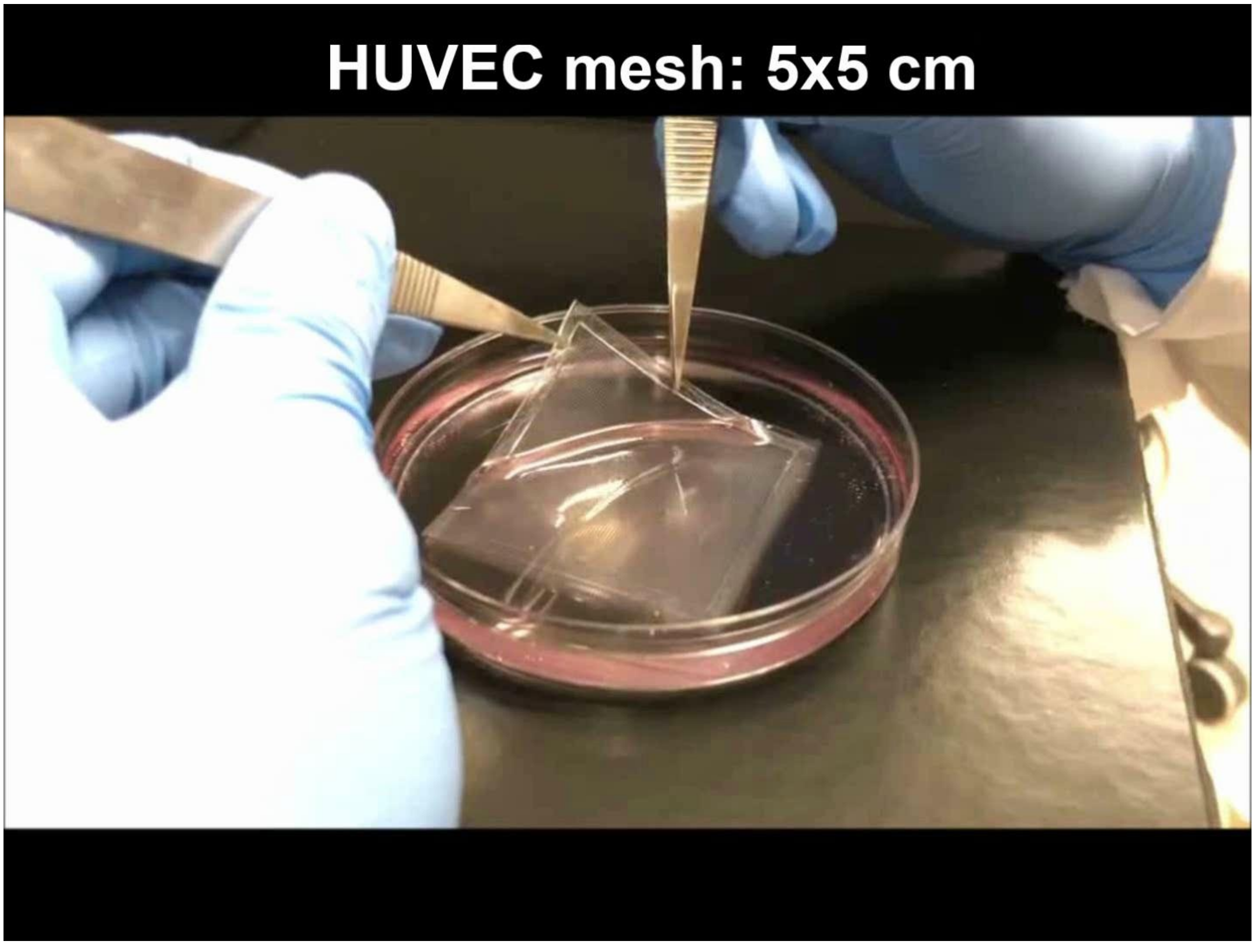

Supplementary Movie 1. A large piece of HUVEC mesh (5×5 cm) is being lifted up from micropillar substrate and microvascular mesh can be easily transferred to other devices or substrates.

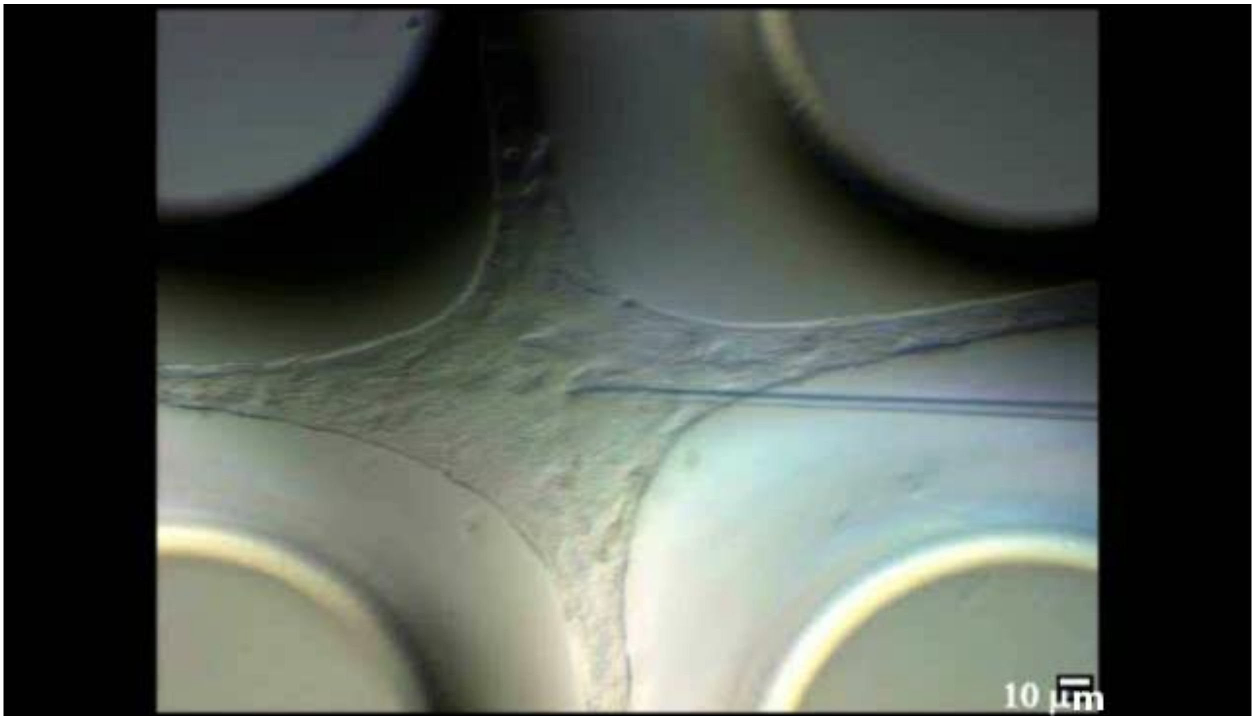

Supplementary Movie 2. A microvascular mesh of HUVEC is being poked using a glass pipette.

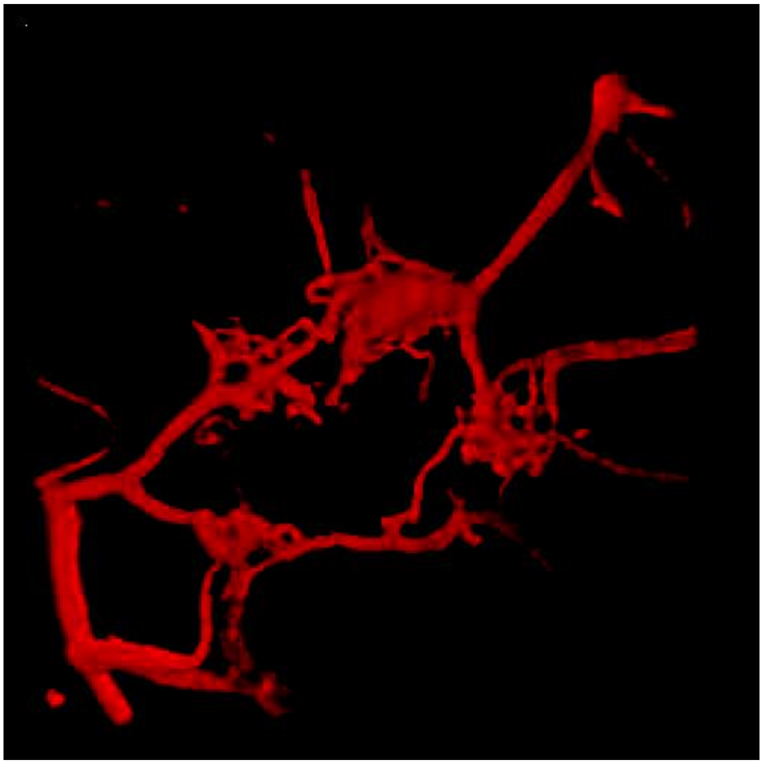

Supplementary Movie 3. 3D structure of several re-vascularized rat islets and anastomoses with host mouse vasculatures in a retrieved HUVEC "Mesh" device after 42 days of transplantation.

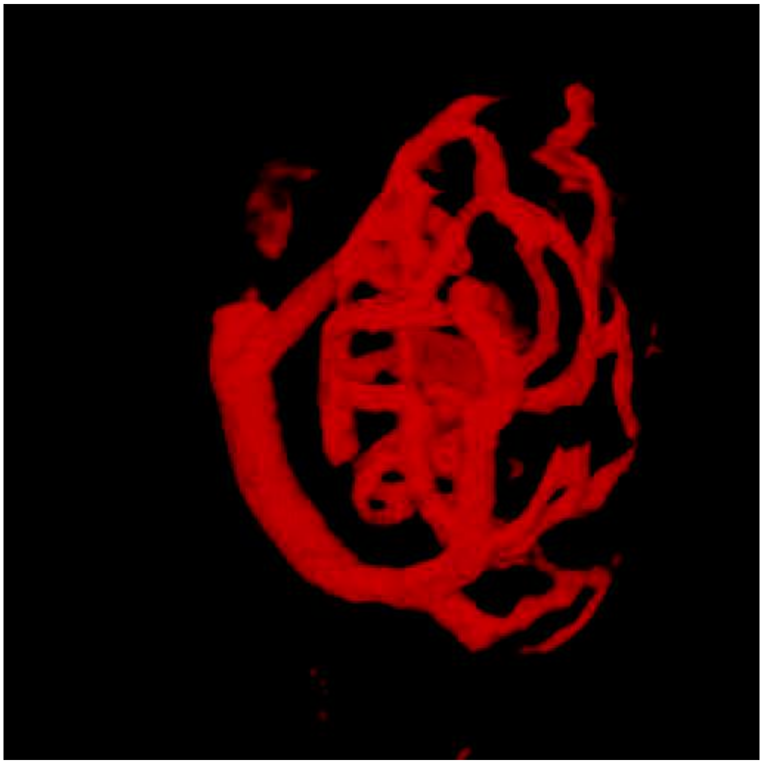

Supplementary Movie 4. 3D structure of a re-vascularized rat islet in a retrieved iPSC-EC Mesh device.
