# Supplementary material for: Engineering transferrable microvascular meshes for subcutaneous islet transplantation
Source: Nat Commun. 2019 Oct 10;10:4602. doi: 10.1038/s41467-019-12373-5 (PMC6787187; doi:10.1038/s41467-019-12373-5)
Supplement: Supplementary file 2 — Description of Additional Supplementary Files [file 41467_2019_12373_MOESM2_ESM.pdf]

Description of Additional Supplementary Files

File Name: Supplementary Movie 1
Description: A large piece of HUVEC mesh (5×5 cm) is being lifted up from micropillar substrate and microvascular mesh can be easily transferred to other devices or substrates.

File Name: Supplementary Movie 2
Description: A microvascular mesh of HUVEC is being poked using a glass pipette.

File Name: Supplementary Movie 3
Description: 3D structure of several re-vascularized rat islets and anastomoses with host mouse vasculatures in a retrieved HUVEC "Mesh" device after 42 days of transplantation.

File Name: Supplementary Movie 4
Description: 3D structure of a re-vascularized rat islet in a retrieved iPSC-EC Mesh device.
