# Supplementary material for: Engineering transferrable microvascular meshes for subcutaneous islet transplantation
Source: Nat Commun. 2019 Oct 10;10:4602. doi: 10.1038/s41467-019-12373-5 (PMC6787187; doi:10.1038/s41467-019-12373-5)
Supplement: Supplementary file 7 — Reporting Summary [file 41467_2019_12373_MOESM7_ESM.pdf]

nature research

Corresponding author(s): Minglin Ma

Last updated by author(s): Aug 28, 2019

Reporting Summary

Nature Research wishes to improve the reproducibility of the work that we publish. This form provides structure for consistency and transparency in reporting. For further information on Nature Research policies, see Authors & Referees and the Editorial Policy Checklist.

Statistics

For all statistical analyses, confirm that the following items are present in the figure legend, table legend, main text, or Methods section.

n/a	Confirmed	
☐	☒	The exact sample size (*n*) for each experimental group/condition, given as a discrete number and unit of measurement
☐	☒	A statement on whether measurements were taken from distinct samples or whether the same sample was measured repeatedly
☐	☒	The statistical test(s) used AND whether they are one- or two-sided<br>*Only common tests should be described solely by name; describe more complex techniques in the Methods section.*
☒	☐	A description of all covariates tested
☐	☒	A description of any assumptions or corrections, such as tests of normality and adjustment for multiple comparisons
☐	☒	A full description of the statistical parameters including central tendency (e.g. means) or other basic estimates (e.g. regression coefficient) AND variation (e.g. standard deviation) or associated estimates of uncertainty (e.g. confidence intervals)
☐	☒	For null hypothesis testing, the test statistic (e.g. *F*, *t*, *r*) with confidence intervals, effect sizes, degrees of freedom and *P* value noted<br>*Give P values as exact values whenever suitable.*
☒	☐	For Bayesian analysis, information on the choice of priors and Markov chain Monte Carlo settings
☒	☐	For hierarchical and complex designs, identification of the appropriate level for tests and full reporting of outcomes
☒	☐	Estimates of effect sizes (e.g. Cohen's *d*, Pearson's *r*), indicating how they were calculated

Our web collection on statistics for biologists contains articles on many of the points above.

Software and code

Policy information about availability of computer code

Data collection	EVOS™ FL Imaging System was used to collected optical, phase-contrast and fluorescence images. Confocal images were captured by FV10-ASW2.0 software (Olympus) under FLUOVIEW FV1000 confocal microscope (Olympus)
Data analysis	GraphPad Prism and R software were used for data plotting and statistical analysis. Fiji ImageJ software was used for image analysis

For manuscripts utilizing custom algorithms or software that are central to the research but not yet described in published literature, software must be made available to editors/reviewers. We strongly encourage code deposition in a community repository (e.g. GitHub). See the Nature Research guidelines for submitting code & software for further information.

Data

Policy information about availability of data

All manuscripts must include a data availability statement. This statement should provide the following information, where applicable:

- Accession codes, unique identifiers, or web links for publicly available datasets
- A list of figures that have associated raw data
- A description of any restrictions on data availability

All relevant data are available within the article and Supplementary Information, and from the corresponding author upon reasonable request.

Field-specific reporting

Please select the one below that is the best fit for your research. If you are not sure, read the appropriate sections before making your selection.

☒ Life sciences ☐ Behavioural & social sciences ☐ Ecological, evolutionary & environmental sciences

For a reference copy of the document with all sections, see nature.com/documents/nr-reporting-summary-flat.pdf

Life sciences study design

All studies must disclose on these points even when the disclosure is negative.

Sample size	Sample size was determined based on previous experience and literatures
Data exclusions	All mice were used for analysis unless they died or had to be euthanized according to IACUC approved protocols.
Replication	Experiments were repeated at least twice. Results were consistent across independent experiments
Randomization	Mice were randomized among control and treatment groups to keep blood glucose levels at the similar level for all groups at the beginning of transplantation
Blinding	Blood glucose measurements were randomly performed by blinded and non-blinded personnel

Reporting for specific materials, systems and methods

We require information from authors about some types of materials, experimental systems and methods used in many studies. Here, indicate whether each material, system or method listed is relevant to your study. If you are not sure if a list item applies to your research, read the appropriate section before selecting a response.

Materials & experimental systems

n/a	Involved in the study	
☐	☒	Antibodies
☐	☒	Eukaryotic cell lines
☒	☐	Palaeontology
☐	☒	Animals and other organisms
☒	☐	Human research participants
☒	☐	Clinical data

Methods

n/a	Involved in the study	
☒	☐	ChIP-seq
☒	☐	Flow cytometry
☒	☐	MRI-based neuroimaging

Antibodies

Antibodies used

Mouse anti-human CD31 (PECAM-1), eBioscience, Cat No 14-0319-82, Clone WM-59
Rabbit anti-human CD31, Sigma, Cat No SAB5600061-100UL, Clone RM247
Goat anti-mouse CD31, R&D systems, Cat No AF3628
Alfa-smooth muscle actin antibody conjugated with Cy3, Sigma, Cat No C6198-.2ML, Clone 1A4
Rabbit anti-rat insulin, abcam, Cat No ab181547, Clone EPR17359
Alexa FluorTM 488 goat anti-mouse antibody, Invitrogen, Cat No A-11001
Alexa FluorTM 555 donkey anti-mouse antibody, Invitrogen, Cat No A-31570
Alexa FluorTM 488 phalloidin, Life technology, Cat No A12379
Texas Red™-X Phalloidin, Life technology, Cat No T7471
Alexa FluorTM 594 donkey anti-rabbit antibody, Invitrogen, Cat No R37119
Alexa FluorTM 488 donkey anti-rabbit antibody, Invitrogen, Cat No R37118
Alexa FluorTM 488 donkey anti-goat antibody, Invitrogen, Cat No A-11055
Alexa FluorTM 594 donkey anti-rabbit antibody, Invitrogen, Cat No R37119

Validation

Mouse anti-human CD31 (PECAM-1), eBioscience, Cat No 14-0319-82, Clone WM-59:
The WM59 monoclonal antibody reacts with human CD31
https://www.thermofisher.com/antibody/product/CD31-PECAM-1-Antibody-clone-WM-59-WM59-Monoclonal/14-0319-82

Rabbit anti-human CD31, Sigma, Cat No SAB5600061-100UL, Clone RM247
This antibody reacts to human CD31 (Platelet endothelial cell adhesion molecule, PECAM-1)
https://www.sigmaaldrich.com/catalog/product/sigma/sab5600061?lang=en&region=US

Goat anti-mouse CD31, R&D systems, Cat No AF3628
Detects mouse and rat CD31/PECAM-1, approximately 10% cross-reactivity with recombinant human CD31 and recombinant porcine CD31 is observed
https://www.rndsystems.com/products/mouse-rat-cd31-pecam-1-antibody_af3628

Alfa-smooth muscle actin antibody conjugated with Cy3, Sigma, Cat No C6198-.2ML, Clone 1A4
Species reactivity: human, mouse, rat, rabbit, guinea pig, chicken, snake, sheep, goat, frog, canine, bovine
Application: immunohistochemistry (formalin-fixed, paraffin-embedded sections)
https://www.sigmaaldrich.com/catalog/product/sigma/c6198?lang=en&region=US

	Rabbit anti-rat insulin, abcam, Cat No ab181547, Clone EPR17359<br>Species reactivity: Mouse, Rat, Human<br>Tested applications, Suitable for: IHC-FoFr, WB, IHC-P, ICC/IF<br>https://www.abcam.com/insulin-antibody-epr17359-ab181547.html

Eukaryotic cell lines

Policy information about cell lines

Cell line source(s)	HUVEC and NHDF were purchased from Lonza
Authentication	Lonza's certificate
Mycoplasma contamination	Cells were not tested for mycoplasma contamination in house but Lonza certified them free of contamination
Commonly misidentified lines<br>(See ICLAC register)	Neither of cell lines are on the ICLAC registry

Animals and other organisms

Policy information about studies involving animals; ARRIVE guidelines recommended for reporting animal research

Laboratory animals	Sprague-Dawley rats (male, strain code 400, 250-275 g weight, Charles River)<br>SD-Tg(UBC-EGFP)2BalRrrc rats (or SD-EGFP, male, 8-12 weeks old, Rat Resource and Research Center)<br>SCID-Beige mice (male and female, model number CBSCBG, 6-8 weeks old, Taconic Biosciences)
Wild animals	The study didn't involve wild animals
Field-collected samples	The study did not involve samples collected from the field
Ethics oversight	All animal procedures were approved by and performed according to the guidelines of the Institutional Animal Care and Use Committee at the Cornell University

Note that full information on the approval of the study protocol must also be provided in the manuscript.
